# The first Pd-catalyzed Buchwald–Hartwig aminations at C-2 or C-4 in the estrone series

**DOI:** 10.3762/bjoc.14.85

**Published:** 2018-05-04

**Authors:** Ildikó Bacsa, Dávid Szemerédi, János Wölfling, Gyula Schneider, Lilla Fekete, Erzsébet Mernyák

**Affiliations:** 1 Department of Organic Chemistry, University of Szeged, Dóm tér 8, H-6720 Szeged, Hungary; 2 Department of Medicinal Chemistry, University of Szeged, Dóm tér 8, H-6720 Szeged, Hungary

**Keywords:** aminoestrones, Buchwald–Hartwig amination, 13α-estrone, functionalization, microwave assisted reactions

## Abstract

A facile Pd-catalyzed C(sp^2^)–N coupling to provide a range of 2- or 4-[(subst.)phenyl]amino-13α-estrone derivatives has been achieved under microwave irradiation. The reactions were mediated with the use of Pd(OAc)_2_ as a catalyst and KO*t-*Bu as a base in the presence of X-Phos as a ligand. The desired products have been obtained in good to excellent yields. The nature and the position of the aniline substituent at the aromatic ring influenced the outcome of the couplings. 2-Amino-13α-estrone was also synthesized in a two-step protocol including an amination of 2-bromo-13α-estrone 3-benzyl ether with benzophenone imine and subsequent hydrogenolysis.

## Introduction

Aminoestrones are of particular interest thanks to their diverse biological applications [1–4]. There exist several aminated steroids in the literature, but the efficient generation of a C(sp^2^)–N bond on the aromatic ring A of estrone derivatives still remains a challenge. Aminoestrones substituted at C-2 or C-4 are mainly produced by the reduction or hydrogenation of the corresponding nitro derivatives [5]. Classical nitration methods have, however, many drawbacks concerning elevated reaction temperatures, long reaction times, and poor yields. The introduction of amino or substituted amino groups onto ring A of estrone is fascinating from both organic chemical and biological points of view. Certain ring A-aminated estrone derivatives are described as inhibitors of estrogen biosynthesis. They are often synthesized via a three-step method including nitration, reduction, and functionalization of the amino group [1–2]. This three-step protocol may be simplified to involve only one or two steps by the application of a Pd-catalyzed Buchwald–Hartwig amination. In recent years, extensive efforts have been made on the Pd(0)-catalyzed amination of aryl halides or triflates in order to achieve the efficient synthesis of substituted anilines [6–9]. Buchwald et al. stated that the Pd source is determining in the amination step [9]. They also found that X-Phos is an outstanding ligand with increased activity and stability compared to those based on BINAP [10].

There are a number of literature methods with respect to microwave-assisted Buchwald–Hartwig couplings [11–13]. Many publications have reported remarkable advantages of microwave-assisted syntheses, including shorter reaction times, higher yields and chemoselectivity [14–16].

Concerning the aromatic ring A of estrone, the Pd-catalyzed Buchwald–Hartwig amination was carried out exclusively at position C-3, starting from the 3-triflate derivative [17–18]. The C(sp^2^)–N cross-coupling of the triflate was achieved with benzophenone imine or benzylamine. The removal of the protecting groups resulted in 3-aminoestrone in high yields. Schön et al. developed two convenient protocols for the preparation of 3-aminoestrone using Pd(OAc)_2_ and Pd_2_(dba)_3_ as catalysts, X-Phos as a ligand, Cs_2_CO_3_ as a base in toluene or DMF solvent under thermal heating or microwave irradiation [18].

We recently described halogenations [19] and Sonogashira couplings on ring A of 13α-estrone and its 3-methyl ether [20]. The 13-epimer of natural estrone is a non-natural C-18 steroid containing *cis* junction of rings C and D [21–22]. This core-modified compound differs from its natural 13β counterpart not only in the configuration of C-13, but also its more flexible conformation. Poirier et al. investigated the in vitro and in vivo estrogenic activity of 3,17-estradiol derivatives of 13α-estrone [23]. The 13-epimers were shown to exhibit no significant binding affinity for estrogen receptor alpha and display no uterotropic activity. Nevertheless, certain 13α-estrone derivatives possess important biological activities including antitumoral effect [24–27]. Thus 13α-estrone is a suitable compound for the development of biologically active steroids lacking estrogenicity. Literature reveals that besides the inversion of C-13, the introduction of an amino group onto C-2 or C-4 of estrone also leads to significant decreases in its binding affinity for nuclear estrogen receptors (ERα and ERβ) [28]. Certain derivatives of 2- or 4-aminoestrone or their 3-methyl ether possess diverse biological activities, including enzyme inhibitory or antiproliferative properties [1–3,29–30]. The 17β-HSD1 enzyme is responsible for the reduction of estrone into 17β-estradiol, which may enhance the proliferation of tumor cells [31]. Effective inhibition of 17β-HSD1 may result in an antitumor effect in hormone-dependent cancers [32]. It is known that several 2- or 4-substituted estrone derivatives possess substantial 17β-HSD1 inhibitory action [19–20,33]. The presence of a large lipophilic group on C-2 of estrone was found to be advantageous concerning the 17β-HSD1 inhibitory activity [33]. Chin et al. reported that 2-bromoacetamidoestrone 3-methyl ether inhibits the 17β-HSD1 enzyme in an irreversible manner [1]. Nevertheless, we proved that certain 4-halogenated 13α-estrone 3-methyl ethers are also effective inhibitors [19]. Recently, we carried out the Pd-catalyzed C–C coupling of 2- and 4-iodo-13α-estrones as well as their 3-methyl ethers with *p*-substituted phenylacetylenes as terminal alkyne partners under microwave irradiation [20]. The regioisomerism markedly influenced the reaction conditions. 2-Iodo isomers were transformed using Pd(PPh_3_)_4_ catalyst and CuI as a cocatalyst. Reactions of the 4-iodo counterparts could be achieved by changing the catalyst to Pd(PPh_3_)_2_Cl_2_ and using higher temperature. Additionally, the 2- or 4-phenylethynyl derivatives were partially or completely saturated in order to get stereochemically different compounds for structure–activity determinations. The saturated derivatives contain a phenyl moiety at C-2 attached through an ethenediyl or ethanediyl linker. Of the synthesized 2- and 4-regioisomers, solely the 2-counterparts bearing a 3-OH group exhibited a substantial inhibitory effect against the 17β-HSD1 enzyme. Surprisingly, the enzyme inhibitory action did not depend on the hybrid state of carbon attached to C-2. From the pharmacological point of view it would be interesting to synthesize and investigate such 13α-estrone derivatives, bearing a lipophilic phenyl group attached to C-2 through an amino linker.

In continuation of our studies with respect to cross-coupling reactions on ring A of 13α-estrone, here we disclose the development of a Pd-catalyzed C(sp^2^)–N coupling methodology for the transformation of 2-bromo- and 4-bromo-13α-estrone 3-methyl (**1** or **3**) as well as 3-benzyl ethers (**2** or **4**) with aniline or substituted anilines as reagents. To the best of our knowledge, there are no literature reports concerning the Pd-catalyzed 2- or 4-amination of the estrane core.

## Results and Discussion

Based on recent literature results [18,20], we started to optimize the reaction conditions for the transformation of 2-bromo-13α-estrone 3-methyl ether (**1**) with aniline (Table 1). Since the Pd source has been shown to be crucial in the amination step, two Pd catalysts were investigated. Namely, Pd(OAc)_2_ and Pd_2_(dba)_3_ were used in the presence of X-Phos or BINAP as ligands. The literature data influenced the selection of the base. The arylation of anilines, escpecially of unsubstituted ones with *o*-bromoanisoles requires stronger bases such as NaO*t-*Bu or KO*t-*Bu [34–38]. This is due to the deactivated, electron-rich nature of anisoles induced by the electron-donating methoxy group. Taking into account the above-mentioned observations [18,34–38], couplings were performed in the presence of DBU, NaO*t-*Bu, KO*t-*Bu or Cs_2_CO_3_ as the base. Toluene was chosen as a solvent, and the reactions were carried out under microwave irradiation or thermal heating. The solvent was selected on the basis of literature data reported for other Pd-catalyzed reactions of estrone derivatives [18,20]. The pre-stirring of the reaction mixture without adding the aryl halide **1** was carried out at 60 °C for 5 min in a water bath, then aryl halide **1** was added and the mixture was irradiated in a microwave reactor at 150 °C for 10 min. The outcome of the couplings greatly depended on the nature of the Pd source, the ligand and the base.

**Table 1:** Effect of the reaction conditions on the Pd-catalyzed amination of 2-bromo-13α-estrone 3-methyl ether (**1**) with aniline in toluene^a^.

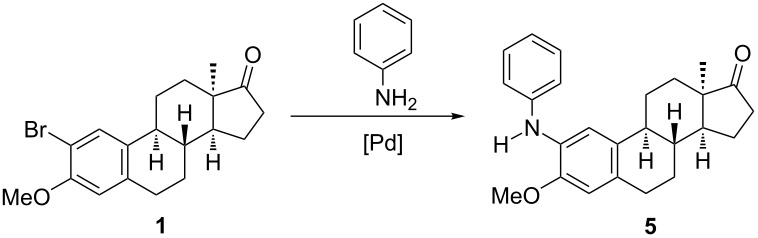						

entry	Pd source (mol %)	ligand (mol %)	base	temp (°C)	**5**^b^ (%)	**5**^c^ (%)

1	Pd(OAc)_2_ (10)	X-Phos, 10	DBU	150	41	44
2	Pd(OAc)_2_ (10)	X-Phos, 10	KO*t-*Bu	150	77	80
3	Pd(OAc)_2_ (10)	X-Phos, 10	KO*t-*Bu	100	83	80
4	Pd(OAc)_2_ (10)	X-Phos, 10	NaO*t-*Bu	150	74	80
5	Pd(OAc)_2_ (10)	X-Phos, 10	NaO*t-*Bu	100	78	81
6	Pd(OAc)_2_ (10)	X-Phos, 10	Cs_2_CO_3_	150	0	0
7	Pd(OAc)_2_ (10)	BINAP, 10	DBU	150	21	22
8	Pd(OAc)_2_ (10)	BINAP, 10	KO*t-*Bu	150	35	37
9	Pd(OAc)_2_ (10)	BINAP, 10	NaO*t-*Bu	150	28	26
10	Pd(OAc)_2_ (10)	BINAP, 10	Cs_2_CO_3_	150	0	0
11	Pd_2_(dba)_3_ (5)	X-Phos, 10	DBU	150	0	0
12	Pd_2_(dba)_3_ (5)	X-Phos, 10	KO*t-*Bu	150	12	12
13	Pd_2_(dba)_3_ (5)	X-Phos, 10	NaO*t-*Bu	150	10	11
14	Pd_2_(dba)_3_ (5)	X-Phos, 10	Cs_2_CO_3_	150	0	0
15	Pd_2_(dba)_3_ (5)	BINAP, 10	DBU	150	0	0
16	Pd_2_(dba)_3_ (5)	BINAP, 10	KO*t-*Bu	150	8	10
17	Pd_2_(dba)_3_ (5)	BINAP, 10	NaO*t-*Bu	150	7	10
18	Pd_2_(dba)_3_ (5)	BINAP, 10	Cs_2_CO_3_	150	0	0

^a^Reagents and conditions: 2-bromo-13α-estrone 3-methyl ether (**1**, 1 equiv), aniline (1.2 equiv). ^b^Flash chromatography yield obtained under conventional heating (24 h, reflux temperature). ^c^Flash chromatography yield obtained under microwave irradiation (10 min).

As summarized in Table 1, reactions with pre-catalyst Pd(OAc)_2_ gave the desired aminoestrone **5** in low to high yields (Table 1, entries 1, 2, 4, 7–9) except when using Cs_2_CO_3_ as the base (Table 1, entries 6 and 10). In the latter cases only dehalogenation of the starting aryl halide was observed in around 20–60% yield. The use of KO*t-*Bu (Table 1, entries 2 and 8) or NaO*t-*Bu (Table 1, entries 4 and 9) as bases seemed to be more advantageous over the use of DBU (Table 1, entries 1 and 7). Concerning the ligand applied, it can be stated that reactions with X-Phos (Table 1, entries 1–6) resulted in higher yields in comparison to couplings with BINAP (Table 1, entries 7–9). As seen in Table 1, our expectations failed concerning couplings induced by Pd_2_(dba)_3_ as the catalyst (Table 1, entries 11–18). None of the Pd_2_(dba)_3_–base–ligand combinations gave compound **5** successfully. Only reactions in the presence of KO*t-*Bu (Table 1, entries 12, 16) or NaO*t-*Bu (Table 1, entries 13, 17) led to the formation of compound **5**, but in very low yields. The starting aryl halide **1** was mostly recovered, and neither dehalogenation nor C(sp^2^)–N coupling occurred.

After finding the best set of reaction conditions (Table 1, entries 2 and 4), the temperature was lowered to 100 °C (Table 1, entries 3 and 5) in order to suppress the dehalogenation side reaction. The efficiency of the couplings was found to be similar to that observed at higher temperature with improved yields. Nevertheless, reaction with KO*t-*Bu (Table 1, entry 3) proved to be slightly more efficient.

In order to compare the efficiency and reaction time of thermal heating with microwave-irradiation conditions, all reactions of **1** with aniline were performed under both conditions (Table 1, entries 1–18). As seen in Table 1, similar yields might be achieved, but reaction times differ considerably (10 min vs 24 h).

On the basis of the optimization procedure discussed above, we selected microwave-assisted conditions at lower temperature (Table 1, entry 3) for further transformations.

**Scheme 1:**
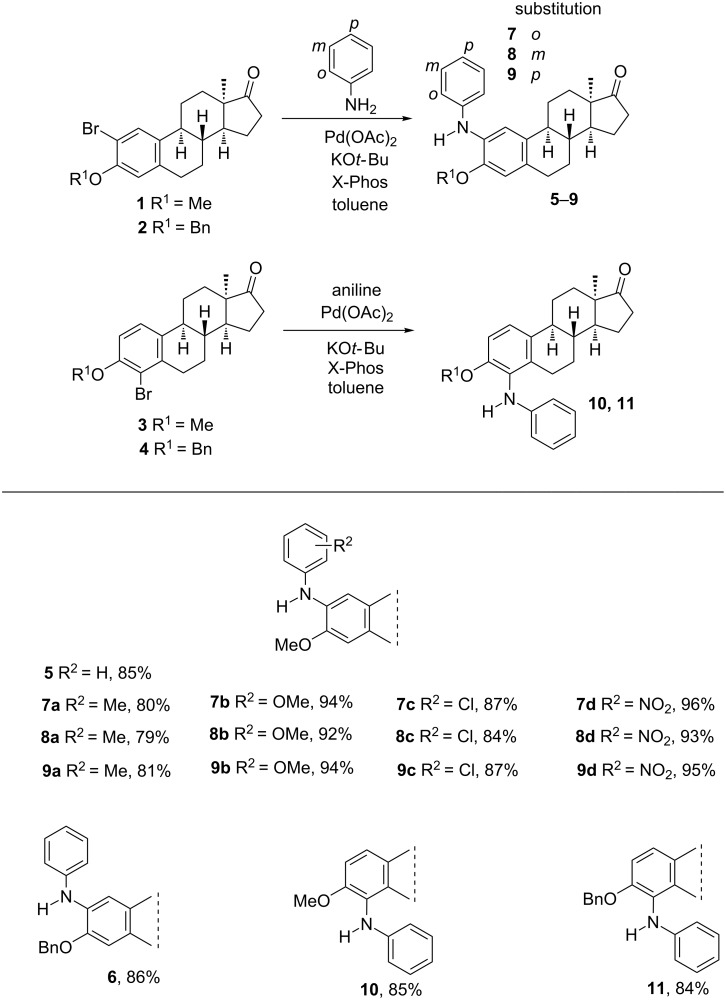
Pd-catalyzed aminations at C-2 or C-4 in the 13α-estrone series. Reactions were performed on a 0.25 mmol scale with 1.2 equiv of amine, 10 mol % Pd(OAc)_2_, 10 mol % X-Phos, at 100 °C, 10 min under microwave irradiation. Flash chromatography yields are reported.

With the best reaction conditions in hand, the couplings at C-2 of starting compound **1** were extended to monosubstituted anilines bearing electronically different substituents at *o*, *m* or *p* positions (Scheme 1).

As indicated in Scheme 1, all couplings proceeded with high yields. The best yields were achieved with nitroanilines, irrespective of the position of the nitro group. Reaction of methylanilines led to slightly lower yields, indicating that the presence of the electron-donating methyl group is less advantageous over the electron-withdrawing nitro function. The coupling at C-4 of compound **1** with aniline under the same conditions yielded aminated derivative **10** in high yield.

With an attempt to investigate the influence of the size of the 3-ether group, 2- or 4-bromo isomers of 3-benzyl ethers **2** or **4** were also submitted to C(sp^2^)–N couplings with aniline using the procedure elaborated above. Irrespective of the more bulky nature of the benzyl ether group compared to its methyl counterpart, compounds **2** and **4** were successfully aminated affording derivatives **6** and **11** without the need of changing the reaction conditions established for couplings at C-2.

**Scheme 2:**
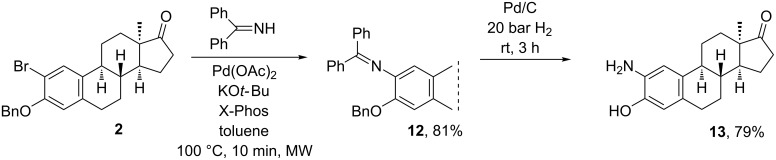
Two-step synthesis of 2-amino-13α-estra-1,3,5(10)-trien-17-one (**13**).

In continuation of our earlier work concerning the synthesis of 2-substituted 3-hydroxy-13α-estrone derivatives as potential enzyme inhibitors [20], here we were interested in the synthesis of 2-amino-13α-estrone (**13**). The efficient C(sp^2^)–N coupling method elaborated above proved to be suitable for the reaction of 2-bromo-3-benzyl ether **2** and benzophenone imine as an amine precursor (Scheme 2). The deprotection was achieved by hydrogenolysis using a Pd/C catalyst. The resulting newly-synthesized 2-amino-13α-estrone (**13**) itself may possess promising pharmacological properties or may serve as a key intermediate in the synthesis of biologically active 2-(subst.)amino-13α-estrones.

The structures of the newly synthesized phenylamino derivatives **5**–**13** were established through ^1^H, ^13^C, HSQC and/or HMBC measurements.

## Conclusion

In conclusion, we have developed a convenient microwave-assisted one-step protocol for the facile and efficient preparation of 2- and 4-phenylaminoestrones **5**–**11**. Our method affords the desired products in short reaction times in good to excellent yields. Thanks to the elaborated mild coupling procedure, the synthesis of 2-amino-13α-estrone **13** could be achieved in only two steps without the first, aromatic nitration step used extensively earlier. The newly synthesized amino derivatives of 13α-estrone **5**–**13** may possess important biological activities without hormonal effect.

## Supporting Information

File 1 Experimental procedures for compounds **5**–**13**, their ^1^H, ^13^C NMR, MS, elemental analysis data and the copies of their ^1^H and ^13^C NMR spectra.

## References

[1] Chin C-C, Asmar P, Warren J C (1980). J Biol Chem.

[2] Bayer Healthcare AG WO patent.

[3] Lawrence Woo L W, Leblond B, Purohit A, Potter B V L (2012). Bioorg Med Chem.

[4] Perreault M, Maltais R, Roy J, Dutour R, Poirier P (2017). ChemMedChem.

[5] Numazawa M, Kimura K (1983). Steroids.

[6] Guram A S, Buchwald S L (1994). J Am Chem Soc.

[7] Paul F, Patt J, Hartwig J F (1994). J Am Chem Soc.

[8] Driver M S, Hartwig J F (1997). J Am Chem Soc.

[9] Wolfe J P, Buchwald S L (2000). J Org Chem.

[10] Strieter E R, Blackmond D G, Buchwald S L (2003). J Am Chem Soc.

[11] Weigand K, Pelka S (2003). Mol Diversity.

[12] Maes B U W, Loones K T J, Lemière G L F, Dommisse R A (2003). Synlett.

[13] Wan Y, Alterman M, Hallberg A (2002). Synthesis.

[14] Kappe C O, Stadler A (2005). Microwaves in Organic and Medicinal Chemistry.

[15] Tierney J P, Lidström P (2009). Microwave Assisted Organic Synthesis.

[16] Kappe C O (2004). Angew Chem, Int Ed.

[17] Radu I-I, Poirier D, Provencher L (2002). Tetrahedron Lett.

[18] Schön U, Messinger J, Buchholz M, Reinecker U, Thole H, Prabhu M K S, Konda A (2005). Tetrahedron Lett.

[19] Bacsa I, Jójárt R, Schneider G, Wölfling J, Maróti P, Herman B E, Szécsi M, Mernyák E (2015). Steroids.

[20] Bacsa I, Jójárt R, Wölfling J, Schneider G, Herman B E, Szécsi M, Mernyák E (2017). Beilstein J Org Chem.

[21] Yaremenko F G, Khvat A V (1994). Mendeleev Commun.

[22] Schönecker B, Lange C, Kötteritzsch M, Günther W, Weston J, Anders E, Görls H (2000). J Org Chem.

[23] Ayan D, Roy J, Maltais R, Poirier D (2011). J Steroid Biochem Mol Biol.

[24] Herman B E, Szabó J, Bacsa I, Wölfling J, Schneider G, Bálint M, Hetényi C, Mernyák E, Szécsi M (2016). J Enzyme Inhib Med Chem.

[25] Mernyák E, Kovács I, Minorics R, Sere P, Czégány D, Sinka I, Wölfling J, Schneider G, Újfaludi Z, Boros I (2015). J Steroid Biochem Mol Biol.

[26] Szabó J, Jerkovics N, Schneider G, Wölfling J, Bózsity N, Minorics R, Zupkó I, Mernyák E (2016). Molecules.

[27] Szabó J, Pataki Z, Wölfling J, Schneider G, Bózsity N, Minorics R, Zupkó I, Mernyák E (2016). Steroids.

[28] Zhu B T, Han G-Z, Shim J-Y, Wen Y, Jiang X-R (2006). Endocrinology.

[29] Fuentes-Aguilar A, Romero-Hernández L L, Arenas-González A, Merino-Montiel P, Montiel-Smith A, Meza-Reyes S, Vega-Báez J L, Plata G B, Padrón J M, López Ó (2017). Org Biomol Chem.

[30] Omar A-M M E, Aboulwafa O M, Leclercq G (1984). J Pharm Sci.

[31] Penning T M (2011). J Steroid Biochem Mol Biol.

[32] Marchais-Oberwinkler S, Henn C, Möller G, Klein T, Negri M, Oster A, Spadaro A, Werth R, Wetzel M, Xu K (2011). J Steroid Biochem Mol Biol.

[33] Möller G, Deluca D, Gege C, Rosinus A, Kowalik D, Peters O, Droescher P, Elger W, Adamski J, Hillisch A (2009). Bioorg Med Chem Lett.

[34] Topchiy M A, Dzhevakov P B, Rubina M S, Morozov O S, Asachenko A F, Nechaev M S (2016). Eur J Org Chem.

[35] Hill L L, Crowell J L, Tutwiler S L, Massie N L, Hines C C, Griffin S T, Rogers R D, Shaughnessy K H, Grasa G A, Johansson Seechurn C C C (2010). J Org Chem.

[36] Ernst J B, Schwermann C, Yokota G-i, Tada M, Muratsugu S, Doltsinis N L, Glorius F (2017). J Am Chem Soc.

[37] Ibrahim H, Bala M D (2016). New J Chem.

[38] Gajare A S, Toyota K, Yoshifuji M, Ozawa F (2004). J Org Chem.

