# Supplementary material for: The first Pd-catalyzed Buchwald–Hartwig aminations at C-2 or C-4 in the estrone series
Source: Beilstein J Org Chem. 2018 May 4;14:998–1003. doi: 10.3762/bjoc.14.85 (PMC6009172; doi:10.3762/bjoc.14.85)
Supplement: File 1 — Experimental procedures for compounds 5–13, their 1H, 13C NMR, MS, elemental analysis data and the copies of their 1H and 13C NMR spectra. [file Beilstein_J_Org_Chem-14-998-s001.pdf]

Supporting Information

for

The first Pd-catalyzed Buchwald–Hartwig aminations at C-2 or C-4 in the estrone series

Ildikó Bacsa[1], Dávid Szemerédi[1], János Wölfling[1], Gyula Schneider[1], Lilla Fekete[2], Erzsébet Mernyák[*,1]

Address: [1]Department of Organic Chemistry, University of Szeged, Dóm tér 8, H-6720 Szeged, Hungary and [2]Department of Medicinal Chemistry, University of Szeged, Dóm tér 8, H-6720 Szeged, Hungary

Email: Erzsébet Mernyák - bobe@chem.u-szeged.hu

*Corresponding author

Experimental procedures for compounds 5–13, their ^{1}H, ^{13}C NMR, MS, elemental analysis data and the copies of their ^{1}H and ^{13}C NMR spectra

Table of contents

Experimental S2

General S2

General procedure for microwave-assisted Pd-catalyzed amination of bromoestrone derivatives (**1**–**4**) S3

General procedure for Pd-catalyzed amination of bromoestrone derivatives (**1**–**4**) by thermal heating S3

3-Methoxy-2-(*N*-phenylamino)-13α-estra-1,3,5(10)-trien-17-one (**5**) S4

3-Benzyloxy-2-(*N*-phenylamino)-13α-estra-1,3,5(10)-trien-17-one (**6**) S4

3-Methoxy-2-(*N*-2’-methylphenylamino)-13α-estra-1,3,5(10)-trien-17-one (**7a**) S5

3-Methoxy-2-(*N*-2’-methoxyphenylamino)-13α-estra-1,3,5(10)-trien-17-one (**7b**) S6

2-(*N*-2’-Chlorophenylamino)-3-methoxy-13α-estra-1,3,5(10)-trien-17-one (**7c**) S6

3-Methoxy-2-(*N*-2’-nitrophenylamino)-13α-estra-1,3,5(10)-trien-17-one (**7d**) S7

3-Methoxy-2-(*N*-3’-methylphenylamino)-13α-estra-1,3,5(10)-trien-17-one (**8a**)............ S7

3-Methoxy-2-(*N*-3’-methoxyphenylamino)-13α-estra-1,3,5(10)-trien-17-one (**8b**)......... S8

2-(*N*-3’-Chlorophenylamino)-3-methoxy-13α-estra-1,3,5(10)-trien-17-one (**8c**)............. S8

3-Methoxy-2-(*N*-3’-nitrophenylamino)-13α-estra-1,3,5(10)-trien-17-one (**8d**) S9

3-Methoxy-2-(*N*-4’-methylphenylamino)-13α-estra-1,3,5(10)-trien-17-one (**9a**).......... S10

3-Methoxy-2-(*N*-4’-methoxyphenylamino)-13α-estra-1,3,5(10)-trien-17-one (**9b**)....... S10

2-(*N*-4’-Chlorophenylamino)-3-methoxy-13α-estra-1,3,5(10)-trien-17-one (**9c**)........... S11

3-Methoxy-2-(*N*-4’-nitrophenylamino)-13α-estra-1,3,5(10)-trien-17-one (**9d**) S11

3-Methoxy-4-(*N*-phenylamino)-13α-estra-1,3,5(10)-trien-17-one (**10**).......................... S12

3-Benzyloxy-4-(*N*-phenylamino)-13α-estra-1,3,5(10)-trien-17-one (**11**)....................... S12

Synthesis of 2-(*N*-diphenylmethylideneamino)-3-benzyloxy-13α-estra-1,3,5(10)-trien-17-one (**12**) S13

Synthesis of 2-amino-13α-estra-1,3,5(10)-trien-17-one (**13**)... S14

Experimental

General

Melting points (mp) were determined with a Kofler hot-stage apparatus and are uncorrected. Elemental analyses were performed with a Perkin-Elmer CHN analyzer model 2400. Thin-layer chromatography: silica gel 60 F_{254}; layer thickness 0.2 mm (Merck); eluent: 30% EtOAc/hexane if otherwise not stated, detection with I_2 or UV (365 nm) after spraying with 5% phosphomolybdic acid in 50% aqueous phosphoric acid and heating at 100–120 °C for 10 min. Flash chromatography: silica gel 60, 40–63 μm (Merck). Reactions under microwave irradiation were carried out with a CEM Corporation focused microwave system, Model Discover SP. The maximum power of irradiation was 200 W. ^{1}H NMR spectra were recorded in DMSO-d_6, $CDCl_3$ solution with a Bruker DRX-500 instrument at 500 MHz, with Me_4Si as

internal standard. ^{13}C NMR spectra were recorded with the same instrument at 125 MHz under the same conditions.The full names of certain chemicals used:

$Pd_2(dba)_3$*: Tris(dibenzylideneacetone)dipalladium(0)X-Phos: 2-Dicyclohexylphosphino-2′,4′,6′-triisopropylbiphenylBINAP: (*R*)-(+)-(1,1′-Binaphthalene-2,2′-diyl)bis(diphenylphosphine)*DBU**:** 1,8-Diazabicyclo[5.4.0]undec-7-ene

Mass spectrometry: Full scan mass spectra of the compounds were acquired in the range of 50 to 1000 *m/z* with a Finnigan TSQ-7000 triple quadrupole mass spectrometer (Finnigan-MAT, San Jose, CA) equipped with a Finnigan electrospray ionization source. Analyses were performed in positive ion mode using flow injection mass spectrometry with a mobile phase of 50% aqueous acetonitrile containing 0.1 v/v% formic acid. The flow rate was 0.3 ml/min. A five µl aliquot of the samples were loaded into the flow. The ESI capillary was adjusted to 4.5 kV and N_2 was used as a nebulizer gas.

*General procedure for microwave-assisted Pd-catalyzed amination of bromoestrone derivatives (**1**–**4**)*

Palladium acetate (5.6 mg, 0.025 mmol, 10 mol %), potassium *tert*-butoxide (56 mg, 0.5 mmol, 2 equiv), X-Phos (12 mg, 0.025 mmol, 10 mol%), aniline (0.30 mmol, 1.2 equiv) and toluene (5 ml) were added into a 10 ml Pyrex pressure vessel (CEM, Part #: 908035) with silicone cap (CEM, Part #: 909210) under nitrogen atmosphere. The mixture was stirred at 60 °C for 5 min and cooled to rt. Then, the 2- or 4-bromoestrone 3-methyl ether (**1** or **3**, 91 mg, 0.25 mmol) or 2- or 4-bromoestrone 3-benzyl ether (**2** or **4**, 110 mg, 0.25 mmol) was charged into the tube, purged with nitrogen and the mixture was heated in a CEM microwave reactor at 100 °C for 10 min under stirring. The solvent was evaporated in vacuo and the residue was purified by flash chromatography with 15% ethyl acetate/85% hexane as eluent.

*General procedure for Pd-catalyzed amination of bromoestrone derivatives (**1**–**4**) by thermal heating*

Palladium acetate (5.6 mg, 0.025 mmol, 10 mol %), potassium *tert*-butoxide (56 mg, 0.5 mmol, 2 equiv.), X-Phos (12 mg, 0.025 mmol, 10 mol %), aniline (0.30 mmol, 1.2 equiv.) and toluene (5 ml) were mixed under nitrogen. The mixture was stirred at 60 °C for 5 min and cooled to rt. Then, the 2- or 4-bromoestrone 3-methyl ether (**1** or **3**, 91 mg, 0.25 mmol) or 2- or 4-bromoestrone 3-benzyl ether (**2** or **4**, 110 mg, 0.25 mmol) was added, and the mixture

was stirred at reflux temperature for 24 h under nitrogen atmosphere. The solvent was evaporated in vacuo and the residue was purified by flash chromatography with 15% ethyl acetate/85% hexane as eluent.

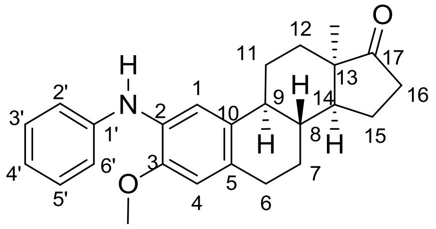

*3-Methoxy-2-(N-phenylamino)-13α-estra-1,3,5(10)-trien-17-one (**5**)*

As described above bromo compound **1** (91 mg, 0.25 mmol) was reacted with aniline (27 µl, 0.30 mmol). After purification by flash chromatography compound **5** was obtained as a white solid (79 mg, 85%, Mp.: 174−176°C, R_f = 0.67). Anal calcd. for $C_{25}H_{29}NO_2$: C, 79.96; H, 7.78. Found: C, 80.05; H, 7.62. M_r: 375.22. ^{1}H NMR (500 MHz, DMSO-d_6) δ ppm :0.96(s, 3H, 18-H_3); 2.74(s, 2H, 6-H_2); 3.73(s, 3H, 3-OCH_3); 6.67(s; 1H, 4-H); 6.69(t, 1H, 4'-H); 6.88(d, 2H, 2'-H, 6'-H); 7.08(s, 1H, 1-H); 7.12(d, 2H, 3'-H, 5'-H); 7.14(s, 1H, NH); ^{13}C NMR (DMSO-d_6) δ ppm : 20.4; 24.5(C-18); 27.8; 28.2; 29.4; 31.6; 32.8; 40.7; 40.8; 48.4; 49.4(C-13); 55.3(3-OCH_3); 111.5(C-4); 115.4(2C: C-2', C-6'); 116.6(C-1) 118.3(C-4'); 128.8(2C: C-3', C-5'); 129.0(C-2); 129.9 and 131.0(C-5, C-10); 144.8(C-1'); 148.6(C-3); 220.6(C-17). MS m/z (%): 376 (100, $[M + H]^+$).

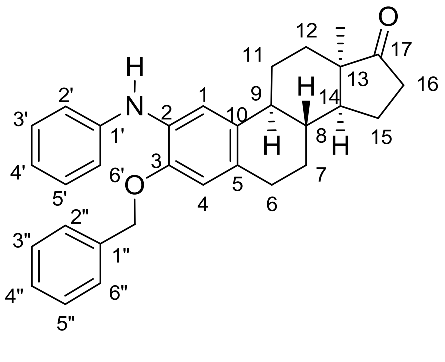

*3-Benzyloxy-2-(N-phenylamino)-13α-estra-1,3,5(10)-trien-17-one (**6**)*

As described above bromo compound **2** (110 mg, 0.25 mmol) was reacted with aniline (27 µl, 0.30 mmol). After purification by flash chromatography compound **6** was obtained as a white solid (97 mg, 86%, Mp.: 75–77°C, R_f =0.64). Anal calcd. for $C_{31}H_{33}NO_2$: C, 82.45; H, 7.37. Found: C, 82.73; H, 7.25. M_r: 451.25 ^{1}H NMR (500 MHz, DMSO-d_6) δ ppm :0.96(s, 3H, 18-

H_3); 2.71(m, 2H, 6-H_2); 5.07(s, 2H, 3-OCH_2); 6.71(t, *J* = 7.2 Hz, 1H, 4'-H); 6.76(s, 1H, 4-H); 6.88(d, *J* = 7.7 Hz, 2H, 2'-H, 6'-H); 7.10(s, 1H, 1-H); 7.13-7.16(overlapping multiplets, 2H, 3'-H, 5'-H, NH); 7.26(m, 1H, 4"-H); 7.31(t, *J* = 7.2 Hz, 2H, 2"-H, 6"-H); 7.36(d, *J* = 7.2 Hz, 2H, 3"-H, 5"-H); ^{13}C NMR (DMSO-d_6) δ ppm: 20.4; 24.5(C-18); 27.8; 29.3; 31.5; 32.8; 40.7; 40.8; 48.4; 49.4(C-13); 69.4(OCH_2); 113.2(C-4); 115.5(2C: C-2', C-6'); 117.6(C-1); 118.3(C-4'); 127.1(2C: C-3", C-5"); 127.4(C-4"); 128.1(2C: C-2", C-6"); 128.7(2C: C-3', C-5'); 129.4 and 130.3(C-2 and C-10); 131.5(C-5); 137.3(C-1"); 145.0(C-1'); 147.8(C-3); 220.6(C-17). MS m/z (%): 452 (100, $[M + H]^+$).

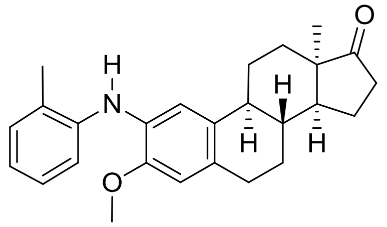

*3-Methoxy-2-(N-2'-methylphenylamino)-13α-estra-1,3,5(10)-trien-17-one (**7a**)*

As described above bromo compound **1** (91 mg, 0.25 mmol) was reacted with 2-methylaniline (33 µl, 0.30 mmol). After purification by flash chromatography compound **7a** was obtained as a white solid (78 mg, 80%, Mp.: 168–170°C, R_f = 0.71). Anal calcd. for $C_{26}H_{31}NO_2$: C, 80.17; H, 8.02. Found: C, 80.37; H, 7.95. M_r: 389.24 ^{1}H NMR (500 MHz, DMSO-d_6) δ ppm :0.95(s, 3H, 18-H_3); 2.15(s, 3H, 2'-Me); 2.75(m, 2H, 6-H_2); 3.75(s, 3H, 3- OCH_3); 6.30(s; 1H, NH); 6.66(s, 1H, 4-H); 6.76(s, 1H, 1-H); 6.80(t, 1H, *J*=7.5 Hz, 5'-H); 6.82(d, 1H, *J*=7.3 Hz, 3 6'-H); 7.04(t, 1H, *J*=7.5 Hz, 4'-H); 7.13(d, 1H, *J*=7.3 Hz, 3'-H); ^{13}C NMR (DMSO-d_6) δ ppm : 17.7(2'-CH_3); 20.4; 24.5(C-18); 27.9; 28.2; 29.3; 30.3; 31.5; 32.8; 40.8; 48.4; 49.4(C-13); 55.4(OCH_3); 111.2(C-4); 115.3(C-1); 117.3(C-6'); 120.3(C-5'); 126.3(C-4') 127.2(C-1'); 129.0(C-2); 130.3(C-3'); 130.4(C-10); 131.0(C-5); 142.3(C-2'); 147.8(C-3); 220.6(C-17). MS m/z (%): 390 (100, $[M + H]^+$).

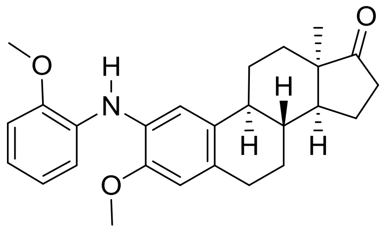

*3-Methoxy-2-(N-2'-methoxyphenylamino)-13α-estra-1,3,5(10)-trien-17-one (**7b**)*

As described above bromo compound **1** (91 mg, 0.25 mmol) was reacted with 2-methoxylaniline (34 µl, 0.30 mmol). After purification by flash chromatography compound **7b** was obtained as a white solid (95 mg, 94%, Mp.: 132–134°C, R_f = 0.53). Anal calcd. for $C_{26}H_{31}NO_3$: C, 77.01; H, 7.71. Found: C, 69.93; H, 7.88. M_r: 405.23 ^{1}H NMR (500 MHz, DMSO-d_6) δ ppm :0.96(s, 3H, 18-H_3); 2.73(m, 2H, 6-H_2); 3.76(s, 3H, 3-OCH_3); 3.82(s, 3H, 3'- OCH_3); 6.37(s; 1H, NH); 6.67(s, 1H, 4-H); 6.77(t, 1H, *J*=7.2 Hz, 4'-H); 6.83(t, 1H, *J*=7.2 Hz, 5'-H); 6.95(d, 1H, *J*=7.9 Hz, 3'-H); 7.02(d, 1H, *J*=7.9 Hz, 6'-H); 7.11(s, 1H, 1-H); ^{13}C NMR (DMSO-d_6) δ ppm : 20.4; 24.5(C-18); 27.8; 28.2; 29.4; 31.6; 32.8; 40.8; 40.9; 48.4; 49.4; 55.5(2C: 3-OCH_3 and 4'-OCH_3); 110.7(C-3'); 111.1(C-4); 113.3(C-6'); 114.5(C-1); 119.1(C-4'); 120.6(C-5'); 128.8(C-10); 129.2(C-2); 131.0(C-5); 132.7(C-1'); 147.3(C-3); 147.8(C-2'); 220.7(C-17). MS m/z (%): 406 (100, $[M + H]^+$).

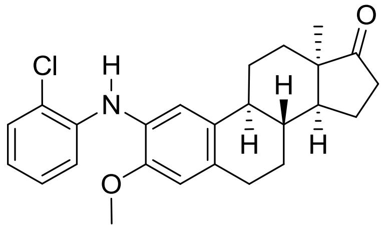

*2-(N-2'-Chlorophenylamino)-3-methoxy-13α-estra-1,3,5(10)-trien-17-one (**7c**)*

As described above bromo compound **1** (91 mg, 0.25 mmol) was reacted with 2-chloroaniline (32 µl, 0.30 mmol). After purification by flash chromatography compound **7c** was obtained as a white solid (89 mg, 87%, Mp.: 70–72°C, R_f = 0.67). Anal calcd. for $C_{25}H_{28}ClNO_2$: C, 73.25; H, 6.88. Found: C, 73.51; H, 6.94. M_r: 409.18 ^{1}H NMR (500 MHz, DMSO-d_6) δ ppm :0.96(s, 3H, 18-H_3); 2.77(m, 2H, 6-H_2); 3.73(s, 3H, 3- OCH_3); 6.64(s; 1H, 4-H); 6.73(s; 1H, NH); 6.74 and 7.12(2t, 2x1H, *J*=7.7 Hz, 4'-H and 5'-H); 6.85 and 7.35(2d, 2x1H, *J*=7.9 Hz, 3'-H and 6'-H); 7.06(s; 1H, 1-H); ^{13}C NMR ($CDCl_3$) δ ppm : 20.1; 25.1(C-18); 28.4; 28.5; 30.1; 32.1; 33.5; 41.6; 41.6; 49.2; 50.2(C-13); 55.7(3-OCH_3); 111.2 and 115.2 and 116.4 and 119.9(C-1 and C-4 and C-4' and C-6'); 121.9(C-10); 127.4(C-5'); 128.6(C-5); 129.6(C-3'); 130.6 and 131.6(C-2 and C-2'); 140.5(C-1'); 148.4(C-3); 221.7(C-17). MS m/z (%): 394 (100); 410 (5, $[M + H]^+$).

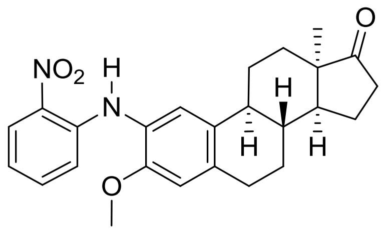

*3-Methoxy-2-(N-2'-nitrophenylamino)-13α-estra-1,3,5(10)-trien-17-one (**7d**)*

As described above bromo compound **1** (91 mg, 0.25 mmol) was reacted with 2-nitroaniline (41 mg, 0.30 mmol). After purification by flash chromatography compound **7d** was obtained as a yellow solid (101 mg, 96%, Mp.: 106–108°C, R_f = 0.55). Anal calcd. for $C_{25}H_{28}N_2O_4$: C, 71.41; H, 6.71. Found: C, 71.32; H, 6.85. M_r: 420.20 ^{1}H NMR (500 MHz, $CDCl_3$) δ ppm :1.06(s, 3H, 18-H_3); 2.86(m, 2H, 6-H_2); 3.80(s, 3H, 3-OCH_3); 6.67(s; 1H, 4-H); 6.72(t, 1H, *J* = 7.1 Hz, 4'-H); 7.11(d, 1H, *J* = 7.9 Hz, 6'-H); 7.34(t, 1H, *J* = 7.1 Hz, 5'-H); 8.18(d, 1H, *J* = 7.9 Hz, 3'-H); 7.25(s; 1H, 1-H); 9.35(s; 1H, NH); ^{13}C NMR ($CDCl_3$) δ ppm : 21.0; 25.0(C-18); 28.3; 28.4; 30.2; 32.0; 33.4; 41.4; 41.4; 49.2; 50.2(C-13); 55.7(3-OCH_3); 111.7(C-4); 116.0(C-6'); 117.0(C-4'); 122.1(C-1); 125.2(C-2); 126.6(C-5'); 132.0(C-10); 133.3(C-2'); 134.8(C-5); 135.5(C-5'); 143.2(C-1'); 151.0(C-3); 221.5(C-17). MS m/z (%): 421 (100, $[M + H]^+$).

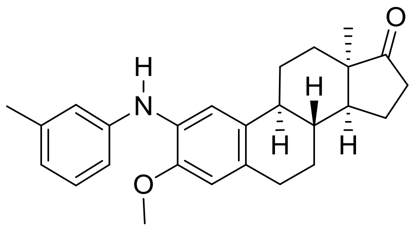

*3-Methoxy-2-(N-3'-methylphenylamino)-13α-estra-1,3,5(10)-trien-17-one (**8a**)*

As described above bromo compound **1** (91 mg, 0.25 mmol) was reacted with 3-methylaniline (32 µl, 0.30 mmol). After purification by flash chromatography compound **8a** was obtained as a white solid (77 mg, 79%, Mp.: 106–107°C, R_f = 0.69). Anal calcd. for $C_{26}H_{31}NO_2$: C, 80.17; H, 8.02. Found: C, 80.38; H, 7.91. M_r: 389.24 ^{1}H NMR (500 MHz, DMSO-d_6) δ ppm : 0.96(s, 3H, 18-H_3); 2.18(s, 3H, 3'-CH_3); 2.74(m, 2H, 6-H_2); 3.73(s, 3H, 3-OCH_3); 6.51(d, 1H, *J*=7.4 Hz, 4'-H); 6.67(s, 1H, 4-H); 6.69(d, 1H, *J*=7.4 Hz, 6'-H); 6.68(s, 1H, 2'-H); 7.01(t, 1H, *J*=7.7 Hz, 5'-H); 7.04(s, 1H, NH); 7.07(s; 1H, 1-H); ^{13}C NMR (DMSO-d_6) δ ppm : 20.4; 21.2(3'-CH_3); 24.5(C-18); 27.8; 28.2; 29.4; 31.6; 32.8; 40.7; 40.8 48.4; 49.4(C-13); 55.3(3-

OCH_3); 111.5(C-4); 112.5(C-6'); 116.2(C-2'); 116.9(C-1); 119.1(C-4'); and 128.6(C-5'); 129.1(C-10); 129.9(C-5); 131.0(C-2); 137.8(C-3'); 144.8(C-1'); 148.6(C-3); 220.6(C-17). MS m/z (%): 390 (100, $[M + H]^+$).

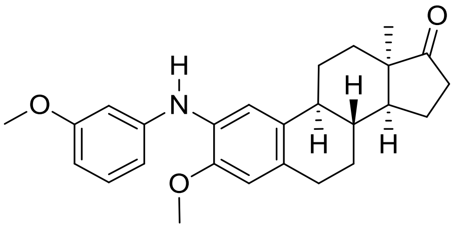

*3-Methoxy-2-(N-3'-methoxyphenylamino)-13α-estra-1,3,5(10)-trien-17-one (**8b**)*

As described above bromo compound **1** (91 mg, 0.25 mmol) was reacted with 3-methoxylaniline (34 µl, 0.30 mmol). After purification by flash chromatography compound **8b** was obtained as a white solid (93 mg, 92%, Mp.: 135–137°C, R_f = 0.51). Anal calcd. for $C_{26}H_{31}NO_3$: C, 77.01; H, 7.71. Found: C, 69.94; H, 7.85. M_r: 405.23 ^{1}H NMR (500 MHz, DMSO-d_6) δ ppm : 0.96(s, 3H, 18-H_3); 2.74(m, 2H, 6-H_2); 3.65(s, 3H, 3-OCH_3); 3.73(s, 3H, 3'-OCH_3); 6.27(m, 1-H, 4'-H); 6.47(m, 2H, 2'-H, 6'-H); 6.50(s, 1H, 4'-H); 6.67(s, 1H, 4-H); 7.03(t, 1H, *J*=7.9 Hz, 5'-H); 7.11(s, 1H, 1-H); 7.17(s; 1H, NH); ^{13}C NMR (DMSO-d_6) δ ppm : 20.4; 24.5(C-18); 27.8; 28.3; 29.4; 31.6; 32.8; 40.7; 40.8; 48.4; 49.4(C-13); 54.6 and 55.3(3-OCH_3 and 3'-OCH_3); 100.8(C-2'); 103.8(C-4'); 107.9(C-6'); 111.5(C-4); 117.1(C-1); 128.8(C-2); 129.5(C-5'); 130.1(C-10); 131.0(C-5); 146.2(C-1'); 148.6(C-3); 160.0(C-3'); 220.7(C-17). MS m/z (%): 406 (100, $[M + H]^+$).

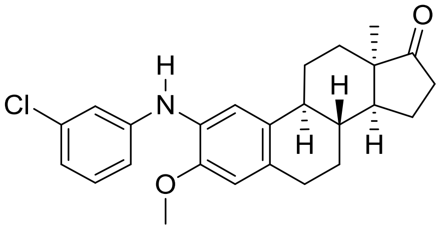

*2-(N-3'-Chlorophenylamino]-3-methoxy-13α-estra-1,3,5(10)-trien-17-one (**8c**)*

As described above bromo compound **1** (91 mg, 0.25 mmol) was reacted with 3-chloroaniline (32 µl, 0.30 mmol). After purification by flash chromatography compound **8c** was obtained as a white solid (86 mg, 84%, Mp.: 72–74°C, R_f = 0.51). Anal calcd. for $C_{25}H_{28}ClNO_2$: C, 73.25; H, 6.88. Found: C, 73.46; H, 6.94. M_r: 409.18 ^{1}H NMR (500 MHz, DMSO-d_6) δ ppm :0.96(s, 3H, 18-H_3); 2.76(m, 2H, 6-H_2); 3.72(s, 3H, 3-OCH_3); 6.65 and 6.74(2d, 2x1H, *J*=6.8 Hz, 4'-

H, 6'-H); 6.71(s; 1H, 4-H); 6.77(s; 1H, 2'-H); 7.07(s, 1H, 1-H); 7.11(t, 1H, *J*=8.0 Hz, 5'-H); 7.55(s, 1H, NH); ^{13}C NMR (DMSO-d_6) δ ppm: 20.4; 24.4(C-18); 27.7; 28.2; 29.4; 31.6; 32.8; 40.7; 40.7; 48.4(C-13); 55.3(3-OCH_3); 111.8(C-4); 112.7(C-6'); 113.7(C-2'); 117.0(C-1); 119.4(C-4'); 127.5(C-2); 130.2(C-5'); 131.3(C-10); 131.9(C-5); 133.3(C-3') 147.2(C-1'); 149.7(C-3); 220.6(C-17). MS m/z (%): 493 (100); 451 (67, $[M + MeCN + H]^+$); 410 (40, $[M + H]^+$).

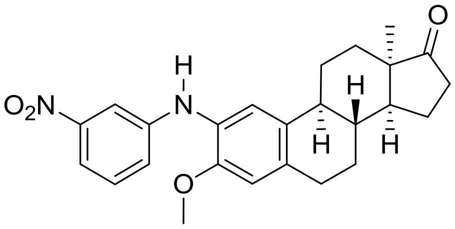

*3-Methoxy-2-(N-3'-nitrophenylamino)-13α-estra-1,3,5(10)-trien-17-one (**8d**)*

As described above bromo compound **1** (91 mg, 0.25 mmol) was reacted with 3-nitroaniline (41 mg, 0.30 mmol). After purification by flash chromatography compound **8d** was obtained as a yellow solid (98 mg, 93%, Mp.: 52–54°C, R_f = 0.47). Anal calcd. for $C_{25}H_{28}N_2O_4$: C, 71.41; H, 6.71. Found: C, 71.33; H, 6.85. M_r: 420.20 ^{1}H NMR (500 MHz, $CDCl_3$) δ ppm :1.06(s, 3H, 18-H_3); 2.83(m, 2H, 6-H_2); 3.82(s, 3H, 3-OCH_3); 6.62(s; 1H, 4-H) 7.22(s; 1H, 1-H); 7.30 and 7.60(2d, 2x1H, *J* = 7.8 Hz, 4'-H and 6'-H); 7.34(t, 1H, *J* = 8.1 Hz, 5'-H); 7.78(s; 1H, NH); ^{13}C NMR ($CDCl_3$) δ ppm : 21.0; 25.1(C-18); 28.3; 28.5; 30.1; 32.0; 33.4; 41.5; 41.6; 49.2; 50.2(C-13); 55.6(3-OCH_3); 110.3 and 111.3 and 114.2 and 116.8 and 121.3(C-1 and C-4 and C-2' and C-4' and C-6'); 127.9(C-10); 129.8(C-5'); 131.6(C-5); 131.9(C-2); 145.5(C-1'); 148.4(C-3); 149.3(C-3'); 221.6(C-17). MS m/z (%): 421 (10, $[M + H]^+$); 453 (100, $[M + MeOH + H]^+$).

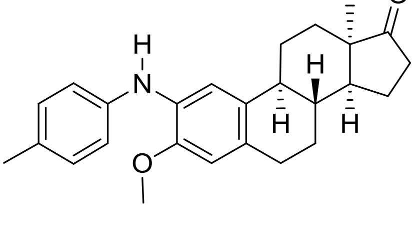

*3-Methoxy-2-(N-4'-methylphenylamino)-13α-estra-1,3,5(10)-trien-17-one (**9a**)*

As described above bromo compound **1** (91 mg, 0.25 mmol) was reacted with 4-methylaniline (32 mg, 0.30 mmol). After purification by flash chromatography compound **9a** was obtained as a white solid (79 mg, 81%, Mp.: 145–147°C, R_f = 0.73). Anal calcd. for $C_{26}H_{31}NO_2$: C, 80.17; H, 8.02. Found: C, 79.88; H, 8.25. M_r: 389.24 ^{1}H NMR (500 MHz, DMSO-d_6) δ ppm :0.95(s, 3H, 18-H_3); 2.18(s, 3H, 4'-CH_3); 2.72(m, 2H, 6-H_2); 3.73(s, 3H, 3-OCH_3); 6.63(s, 1H, 4-H); 6.84(d, 2H, *J* = 8.9 Hz, 2'-H, 6'-H); 6.95(s, 1H, NH); 6.97(d, 2H, *J*=8.9 Hz, 3'-H, 5'-H); 6.95(s, 1H, NH); 7.03(s, 1H, 1-H); ^{13}C NMR (DMSO-d_6) δ ppm : 20.1(4'-CH_3); 20.4; 24.5(C-18); 27.9; 28.2; 29.3; 31.5; 32.8; 40.8; 40.9; 48.4; 49.4(C-13); 55.3(OCH_3); 111.3(C-4); 114.8(C-1); 116.4(2C: C-2', C-6'); 129.2(2C: C-3', C-5'); 127.4(C-1'); 128.9(C-10); 129.8(C-2); 131.0(C-5); 141.8(C-4'); 147.8(C-3); 220.6(C-17). MS m/z (%): 390 (100, $[M + H]^+$).

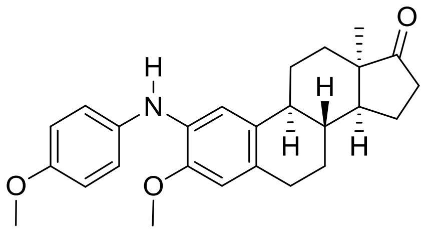

*3-Methoxy-2-(N-4'-methoxyphenylamino)-13α-estra-1,3,5(10)-trien-17-one (**9b**)*

As described above bromo compound **1** (91 mg, 0.25 mmol) was reacted with 4-methoxylaniline (35µl, 0.30 mmol). After purification by flash chromatography compound **9b** was obtained as a white solid (95 mg, 94%, Mp.: 83–85°C, R_f = 0.60). Anal calcd. for $C_{26}H_{31}NO_3$: C, 77.01; H, 7.71. Found: C, 69.85; H, 7.93. M_r: 405.23 ^{1}H NMR (500 MHz, DMSO-d_6) δ ppm : 0.95(s, 3H, 18-H_3); 2.70(m, 2H, 6-H_2); 3.68(s, 3H, 3-OCH_3); 3.74(s, 3H, 3'-OCH_3); 6.61(s, 1H, 4-H); 6.79(s, 1H, NH); 6.80 (d, 2H, *J* = 8.8 Hz, 3'-H, 5'-H); 6.92(s, 1H, 1-H); 6.93(d, 2H, *J* = 8.8 Hz, 2'-H, 6'-H); ^{13}C NMR ($CDCl_3$) δ ppm : 20.4; 24.5(C-18); 27.9; 28.3; 29.3; 31.5; 32.8; 40.8; 40.9; 48.4; 49.4; 55.1 and 55.3(3-OCH_3 and 4'-OCH_3); 111.2(C-4); 112.7(C-1); 114.3(2C: C-3', C-5'); 119.1(2C: C-2', C-6'); 127.7(C-1'); 130.9(C-10); 131.0(C-5); 137.1(C-2); 147.0(C-3); 153.0(C-4'); 220.7(C-17). MS m/z (%): 406 (100, $[M + H]^+$).

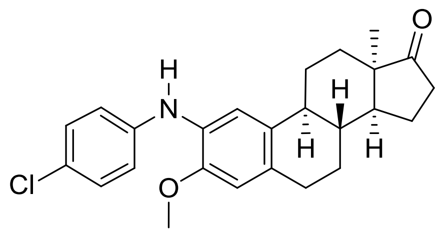

*2-(N-4'-Chlorophenylamino)-3-methoxy-13α-estra-1,3,5(10)-trien-17-one (**9c**)*

As described above bromo compound **1** (91 mg, 0.25 mmol) was reacted with 4-chloroaniline (38 mg, 0.30 mmol). After purification by flash chromatography compound **9c** was obtained as a white solid (89 mg, 87%, Mp.: 188–190°C, R_f = 0.51). Anal calcd. for $C_{25}H_{28}ClNO_2$: C, 73.25; H, 6.88. Found: C, 73.47; H, 7.03. M_r: 409.18 ^{1}H NMR (500 MHz, DMSO-d_6) δ ppm :0.96(s, 3H, 18-H_3); 2.76(m, 2H, 6-H_2); 3.73(s, 3H, 3-OMe); 6.69(s; 1H, 4-H); 6.83(d, 2H, *J* = 8.7 Hz, 2'-H, 6'-H); and 7.14(d, 2H, *J* = 8.7 Hz, 3'-H, 5'-H); 7.06(s; 1H, 1-H); 7.41(s; 1H, NH); ^{13}C NMR (DMSO-d_6) δ ppm : 20.4; 24.5(C-18); 27.8; 28.2; 29.4; 31.6; 32.8; 40.7; 40.8; 48.4; 49.4, 55.3(OCH_3); 111.7(C-4); 116.3(2C: C-2', C-6'); 117.8(C-1); 121.0(C-4'); 128.3(C-2); 128.5(2C: C-3', C-5'); 131.0(C-10); 131.2(C-5); 144.2(C-1'); 149.1(C-3); 220.6(C-17). MS m/z (%): 451 (100, $[M + MeCN + H]^+$); 410 (51, $[M + H]^+$).

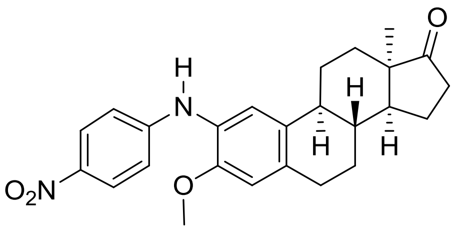

*3-Methoxy-2-(N-4'-nitrophenylamino)-13α-estra-1,3,5(10)-trien-17-one (**9d**)*

As described above bromo compound **1** (91 mg, 0.25 mmol) was reacted with 4-nitroaniline (41 mg, 0.30 mmol). After purification by flash chromatography compound **9d** was obtained as a yellow solid (100 mg, 95%, Mp.: 112–114°C, R_f = 0.35). Anal calcd. for $C_{25}H_{28}N_2O_4$: C, 71.41; H, 6.71. Found: C, 71.58; H, 6.55. M_r: 420.20 ^{1}H NMR (500 MHz, DMSO-d_6) δ ppm :0.96(s, 3H, 18-H_3); 2.80(m, 2H, 6-H_2); 3.72(s, 3H, 3-OCH_3); 6.71(d, 2H, *J* = 9.2 Hz, 2'-H, 6'-H); 6.80(s; 1H, 4-H); 7.11(s; 1H, 1-H); 8.00(d, 2H, *J* = 9.2 Hz, 3'-H, 5'-H); 8.72(s, 1H, NH); ^{13}C NMR (DMSO-d_6) δ ppm : 20.4; 24.5(C-18); 27.7; 28.0; 29.5; 31.5; 32.8; 40.6; 40.7; 48.4; 49.4(C-13); 55.3(OCH_3); 112.1(C-4); 112.4(2C: C-2' and C-6'); 122.5(C-1); 125.2(C-

2); 125.9(2C: C-3' and C-5'); 131.7(C-10); 134.9(C-5); 136.8(C-4'); 151.0(C-1'); 152.6(C-3); 220.6(C-17). MS m/z (%): 421 (7, $[M + H]^+$); 493 (100, $[M + MeCN + MeOH + H]^+$).

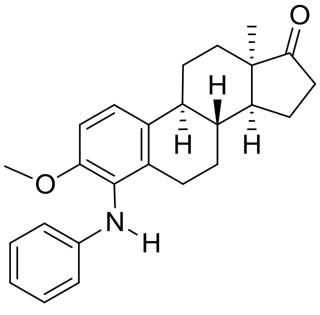

*3-Methoxy-4-(N-phenylamino)-13α-estra-1,3,5(10)-trien-17-one (**10**)*

As described above bromo compound **3** (91 mg, 0.25 mmol) was reacted with aniline (27 µl, 0.30 mmol). After purification by flash chromatography compound **10** was obtained as a white solid (80 mg, 85%, Mp.:180–182°C, R_f =0.60). Anal calcd. for $C_{25}H_{29}NO_2$: C, 79.96; H, 7.78. Found: C, 80.12; H, 7.93. M_r: 375.22 ^{1}H NMR (500 MHz, $CDCl_3$) δ ppm :1.06(s, 3H, 18-H_3); 2.47 and 2.81(2xm, 2H, 6-H_2); 3.77(s, 3H, 3-OMe); 6.65(d, 2H, *J*= 8.3 Hz, 2'-H and 6'-H), 6.79(d, 1H, *J*= 8.6 Hz, 2-H); 6.83(t, 1H, *J*=7.9 Hz, 4'-H); 7.12(d, 1H, *J*=8.6 Hz, 1-H); 7.19(t, 2H, *J*=7.4 Hz, 3'-H and 5'-H); ^{13}C NMR ($CDCl_3$) δ ppm: 20.9; 25.0(C-18); 26.1; 27.9; 28.6; 32.2; 33.4; 41.1; 41.8; 49.4; 50.2(C-13); 55.8(OCH_3); 108.7(C-2); 115.6(2C: C-2', C-6'); 119.6(C-4'); 123.0(C-1); 127.8(C-4); 129.0(2C: C-3', C-5'); 133.2(C-10); 134.5(C-5); 145.5(C-1'); 151.9(C-3); 221.8(C-17). MS m/z (%): 376 (100, $[M + H]^+$).

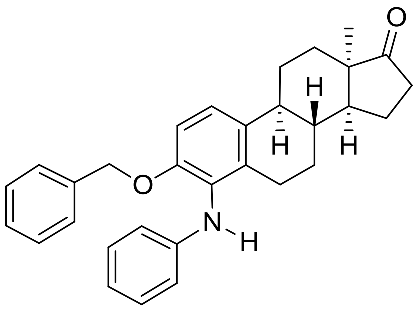

*3-Benzyloxy-4-(N-phenylamino)-13α-estra-1,3,5(10)-trien-17-one (**11**)*

As described above bromo compound **4** (110 mg, 0.25 mmol) was reacted with aniline (27 µl, 0.30 mmol). After purification by flash chromatography compound **11** was obtained as a

white solid (95 mg, 84%, Mp.: 56–58°C, R_f = 0.62). Anal calcd. for $C_{31}H_{33}NO_2$: C, 82.45; H, 7.37. Found: C, 82.63; H, 7.51. M_r: 451.25 ^{1}H NMR (500 MHz, DMSO-d_6) δ ppm :0.97(s, 3H, 18-H_3); 2.30 and 2.75(2xm, 2H, 6-H_2); 5.02(s, 2H, 3-OCH_2);); 6.47(d, 2H, *J* = 7.7 Hz, 2'-H, 6'-H), 6.58(t, 1H, *J* = 7.2 Hz, 4'-H); 6.80(d, 1H, *J* = 8.6 Hz, 2-H); 6.98(s, 1H, NH); 7.05(t, 2H, *J* = 7.7 Hz, 3'-H, 5'-H); 7.08(d, 1H, *J* = 8.6 Hz, 1-H); 7.19-7.24(overlapping multiplets, 5H, 2"-H, 3"-H, 4"-H, 5"-H, 6"-H); ^{13}C NMR (DMSO-d_6) δ ppm: 20.4; 24.4(C-18); 25.5; 27.4; 28.1; 31.6; 32.7; 40.3; 41.1; 48.5; 49.3(C-13); 69.2(OCH_2); 110.9(C-2); 113.1(2C: C-2', C-6'); 116.5(C-4'); 123.0(C-1); 126.9(2C: C-2", C-6"); 127.3(C-4"); 127.8(C-4); 128.0(2C: C-3", C-5"); 128.5(2C: C-3', C-5'); 132.8(C-10); 135.5(C-5); 137.3(C-1"); 147.0(C-1'); 151.8(C-3); 220.6(C-17). MS m/z (%): 452 (100, $[M + H]^+$).

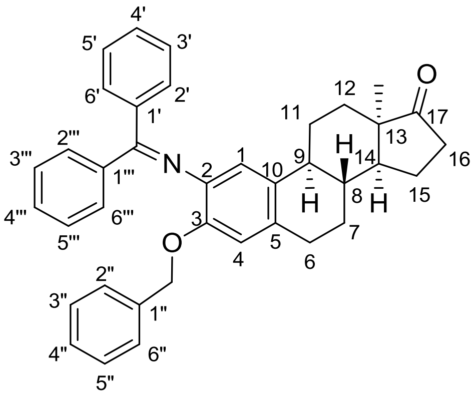

*Synthesis of 3-benzyloxy-2-(N-diphenylmethylideneamino)-13α-estra-1,3,5(10)-trien-17-one **(12)***

Palladium acetate (5.6 mg, 0.025 mmol, 10 mol %), potassium *tert*-butoxide (56 mg, 0.5 mmol, 2 equiv.), X-Phos (12 mg, 0.025 mmol, 10 mol %), benzophenone-imine (50 µl, 0.30 mmol) and toluene (5 ml) were added into a 10 mL Pyrex pressure vessel (CEM, Part #: 908035) with silicone cap (CEM, Part #: 909210) under nitrogen atmosphere. The mixture was stirred at 60 °C for 5 min and cooled to rt. Then, the 3-benzyloxy-2-bromo-13α-estra-1,3,5(10)-trien-17-one (**2**) (110 mg, 0.25 mmol) was charged into the tube, purged with nitrogen and the mixture was heated in a CEM microwave reactor at 100 °C for 30 min under stirring. The solvent was evaporated in vacuo and the residue was purified by flash chromatography with 15% ethyl acetate/85% hexane as eluent. yielding 110 mg (81%) of compound **12**. After purification by flash chromatography compound **12** was obtained as a yellow solid (110 mg, 81 %, Mp.: 60–62°C, R_f = 0.58). Anal calcd. for $C_{38}H_{37}NO_2$, C, 84.57; H, 6.91. Found: C, 84.67; H, 7.08. M_r: 539.28 ^{1}H NMR (500 MHz, DMSO-d_6) δ ppm :0.93(s,

3H, 18-H_3); 2.62(m, 2H, 6-H_2); 4.97(s, 2H, 3-OCH_2); 6.45(s, 1H, 1-H); 6.58(s, 1H, 4-H); 7.07-7.62(overlapping multiplets, 15H, 2'-H, 3'-H, 4'-H, 5'-H, 6'-H; 2''-H, 3''-H, 4''-H, 5''-H, 6''-H; 2'''-H, 3'''-H, 4'''-H, 5'''-H, 6'''-H); ^{13}C NMR (DMSO-d_6) δ ppm: 20.8; 24.9(C-18); 28.1; 28.5; 29.8; 32.1; 33.3; 41.3; 48.8; 49.9(C-13); 69.8(OCH_2); 113.7(C-4); 118.7(C-1); 127.5(2C) and 128.0 and 128.4(2C) and 128.6(2C) and 128.8(3C) and 128.9 and 129.1(2C) and 130.1 and 131.2(C-2', C-3', C-4', C-5', C-6', C-2", C-3", C-4", C-5", C-6", C-2''', C-3''', C-4''', C-5''', C-6'''); 131.8 and 132.2 and 137.0 and 138.1 and 139.1 and 139.4(C-2, C-5, C-10, C-1', C-1", C-1'''); 146.5(C-3); 168.4(C=N); 221.2(C-17). MS m/z (%): 540 (100, $[M + H]^+$).

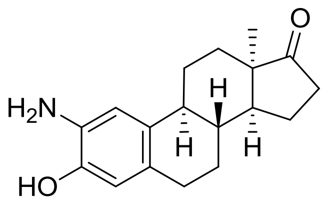

*Synthesis of 2-amino-13α-estra-1,3,5(10)-trien-17-one (**13**)*

A suspension of **12** (100 mg, 0.185 mmol) and Pd/C (0.35 g, 10%) in EtOAc (20 ml) was subjected to 20 bar of H_2 at room temperature for 3 h. The catalyst was then removed by filtration through a short pad of silica gel. After evaporation of the solvent in vacuo, the crude product was subjected to column chromatography with 15% ethyl acetate/85% hexane as eluent to afford compound **13** in 79% yield (53 mg, Mp.: 208–210 °C, R_f = 0.55 (70% EtOAc/hexane)). Anal calcd. for $C_{18}H_{23}NO_2$: C, 75.76; H, 8.12. Found: C, 75.58; H, 8.23. M_r: 285.17. ^{1}H NMR (500 MHz, DMSO-d_6) δ ppm :0.95(s, 3H, 18-H_3); 2.55(m, 2H, 6-H_2); 4.20(broad s, 2H, 2-NH_2); 6.29(s; 1H, 4-H); 6.49(s, 1H, 1-H); 8.66(s, 1H, OH). ^{13}C NMR (DMSO-d_6) δ ppm : 20.4; 24.5(C-18); 28.1; 28.3; 28.9; 31.7; 32.8; 40.9; 41.1; 48.5; 49.4; 111.9 and 114.1 (C-1 and C-4); 124.1 and 129.7(C-5 and C-10); 134.0(C-2); 142.2(C-3); 220.7(C-17). MS m/z (%):286 (100, $[M + H]^+$).

5

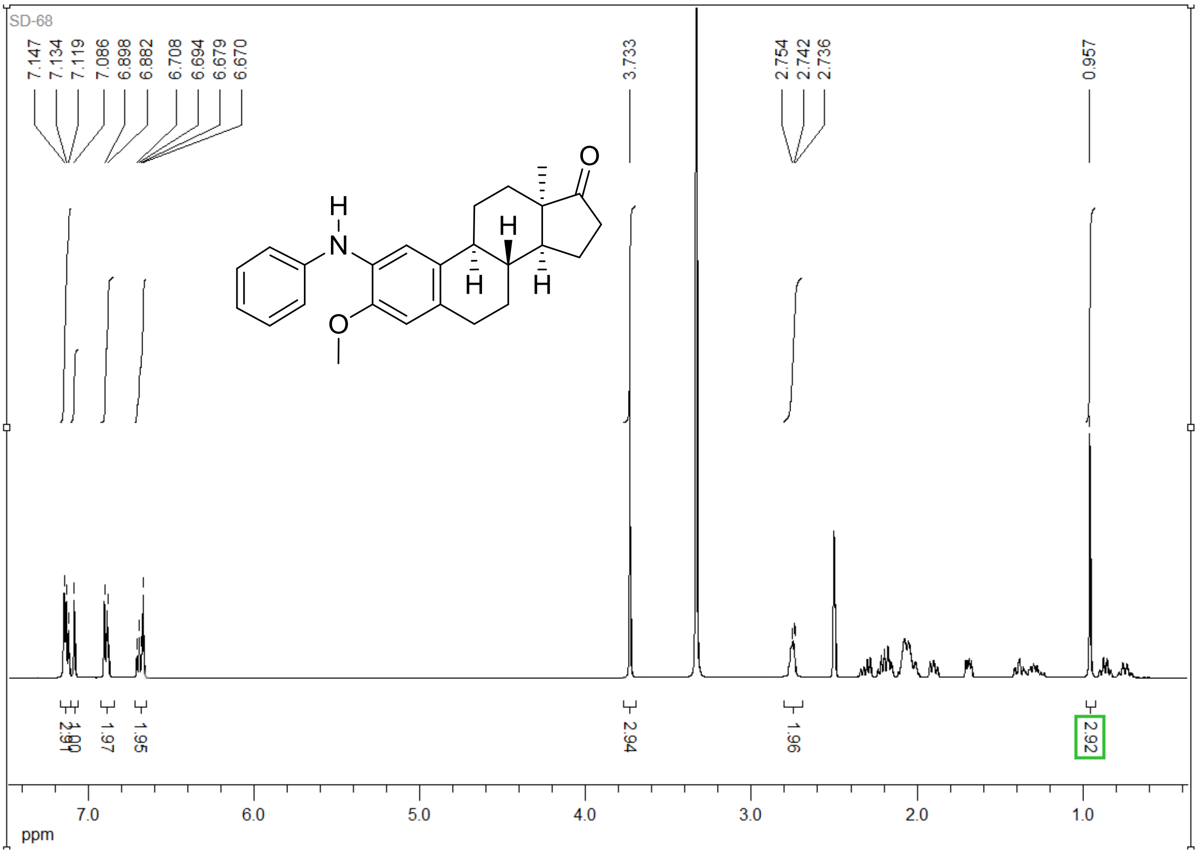
SD-68
7.147
7.134
7.119
7.086
6.898
6.882
6.708
6.694
6.679
6.670
3.733
2.754
2.742
2.736
0.957
H
N
O
H
H
H
O
1.97
1.95
2.94
1.96
2.92
7.0
6.0
5.0
4.0
3.0
2.0
1.0
ppm

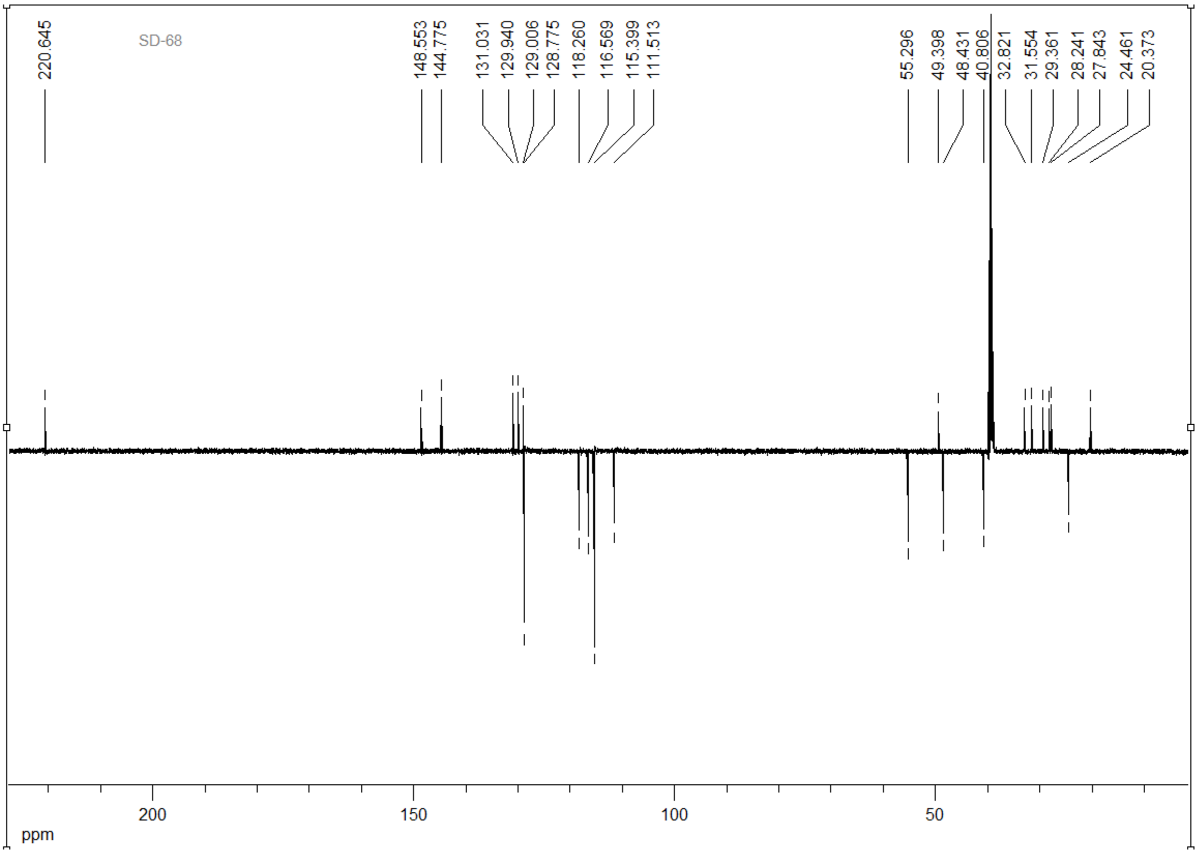
220.645
SD-68
148.553
144.775
131.031
129.940
129.006
128.775
118.260
116.569
115.399
111.513
55.296
49.398
48.431
40.806
32.821
31.554
29.361
28.241
27.843
24.461
20.373
200
150
100
50
ppm

6

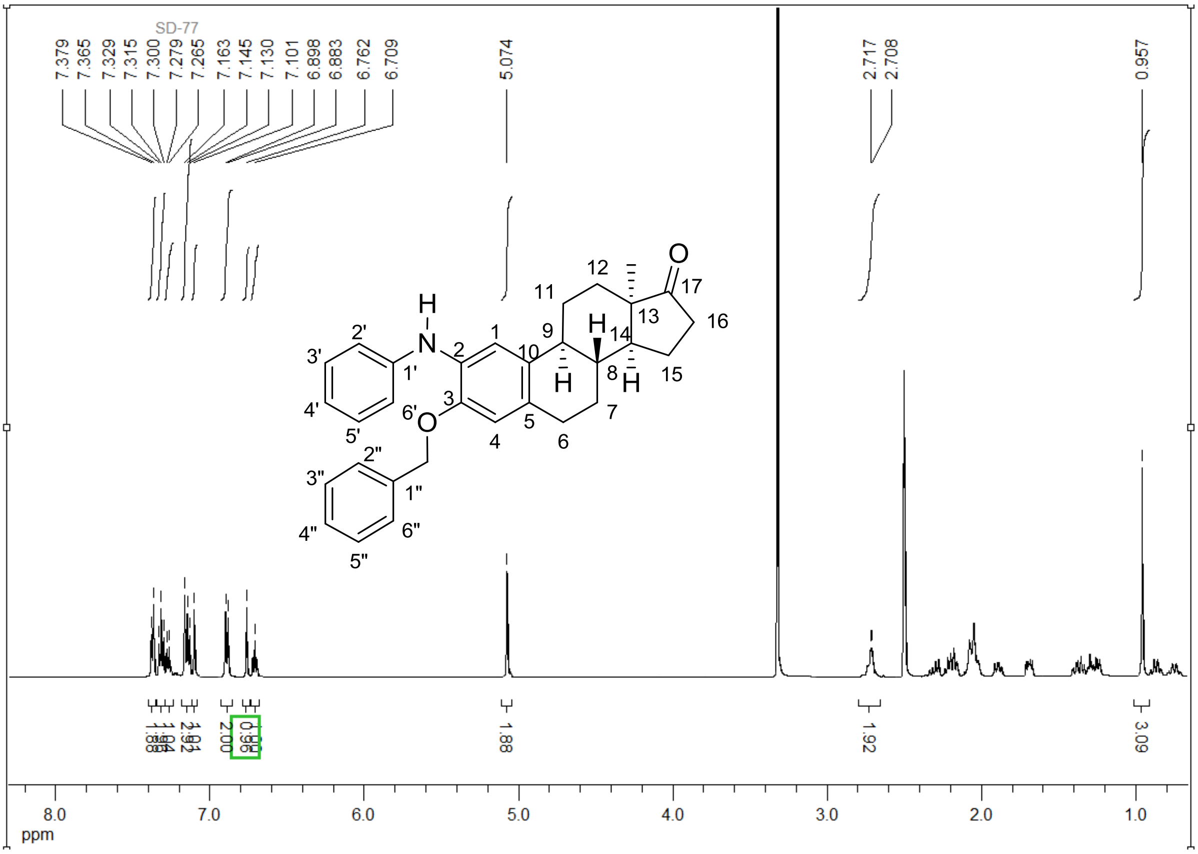

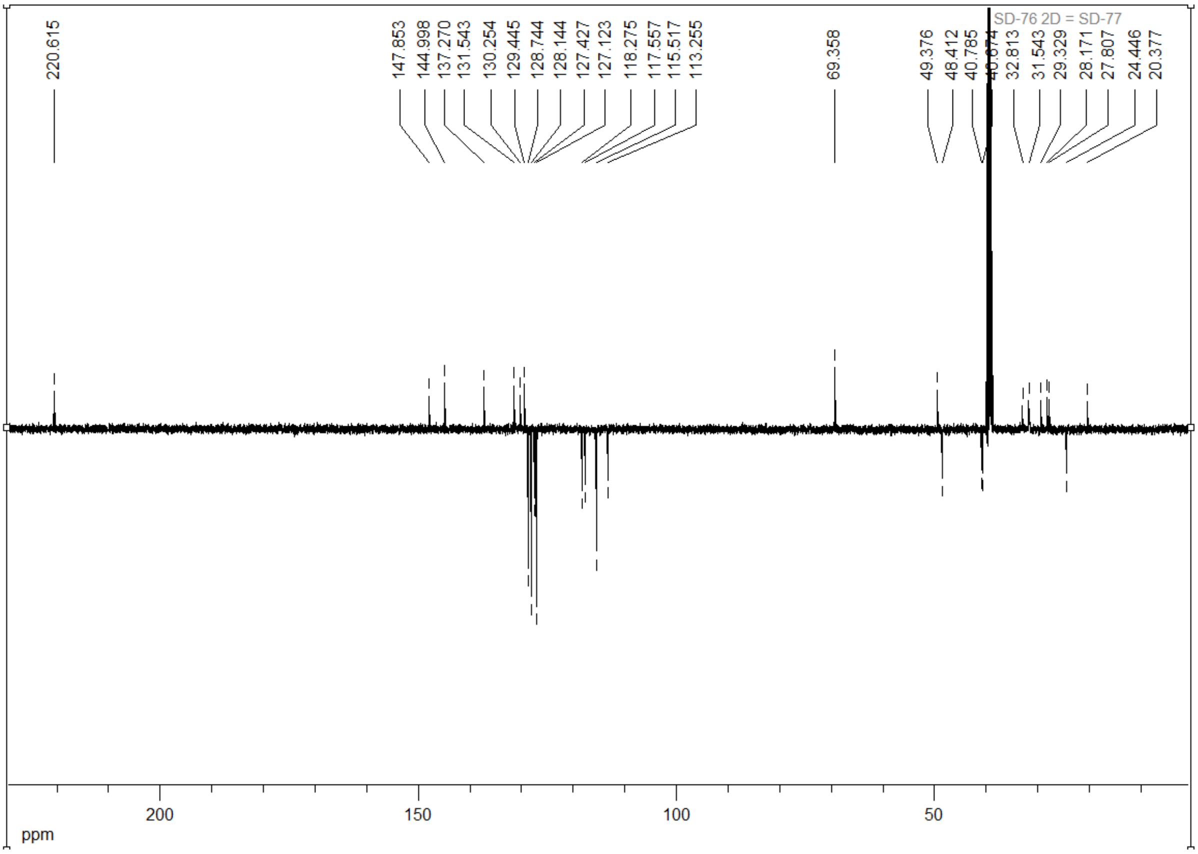

7a

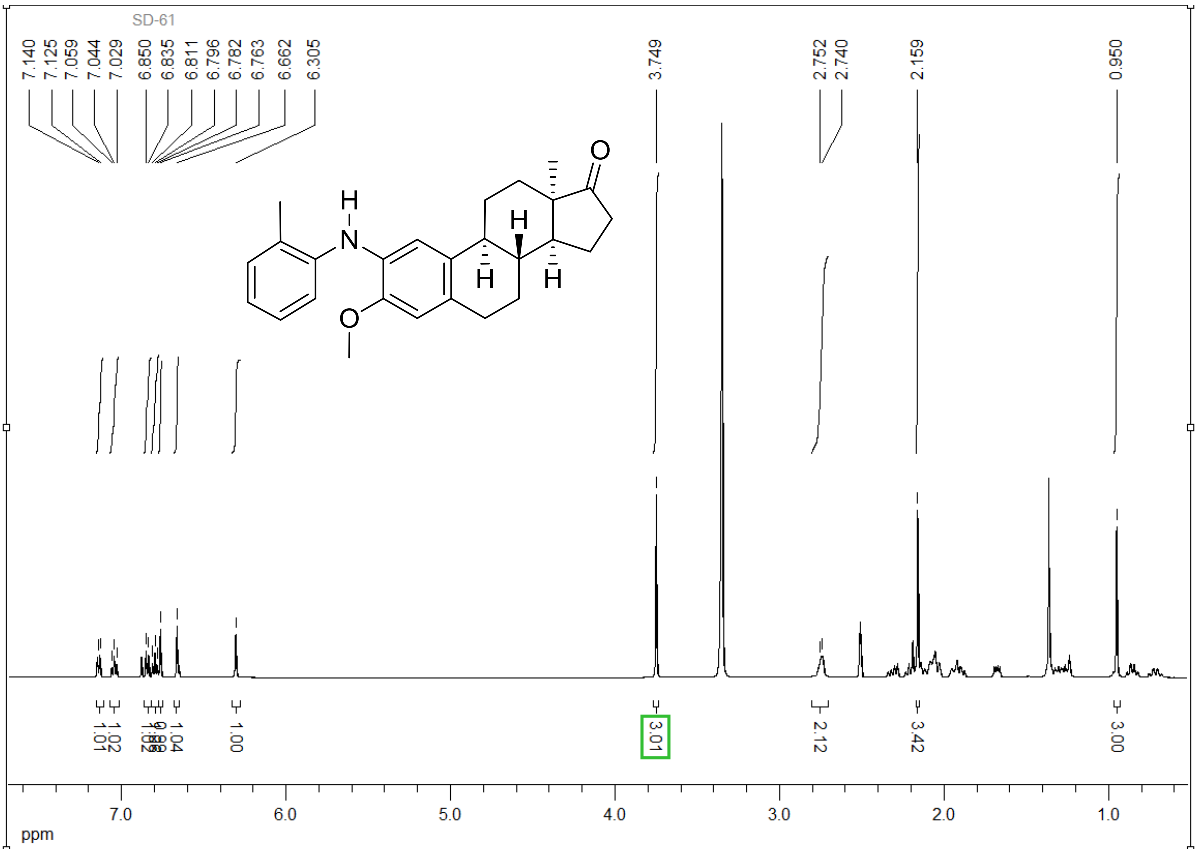
SD-61
7.140
7.125
7.059
7.044
7.029
6.850
6.835
6.811
6.796
6.782
6.763
6.662
6.305
3.749
2.752
2.740
2.159
0.950
H
N
O
H
H
H
O
1.01
1.02
1.04
1.00
3.01
2.12
3.42
3.00
7.0
6.0
5.0
4.0
3.0
2.0
1.0
ppm

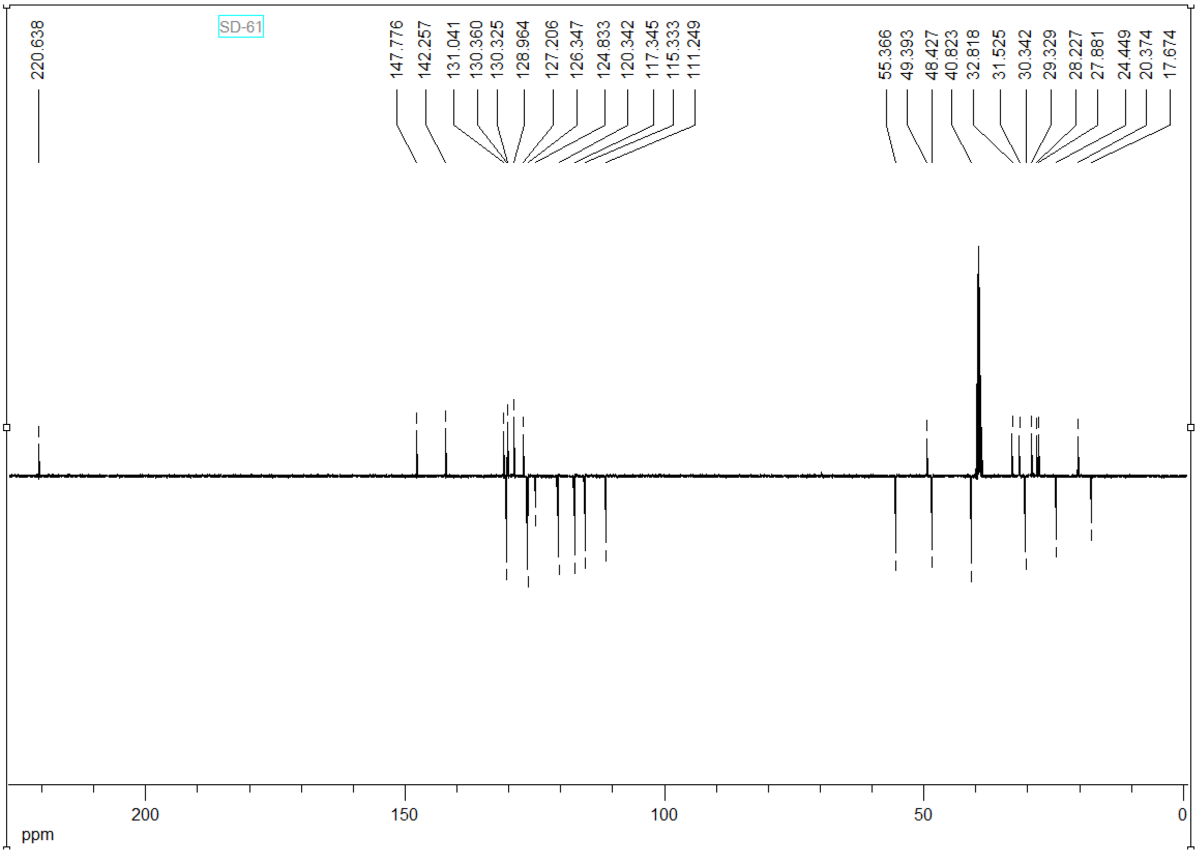
SD-61
220.638
147.776
142.257
131.041
130.360
130.325
128.964
127.206
126.347
124.833
120.342
117.345
115.333
111.249
55.366
49.393
48.427
40.823
32.818
31.525
30.342
29.329
28.227
27.881
24.449
20.374
17.674
200
150
100
50
0
ppm

7b

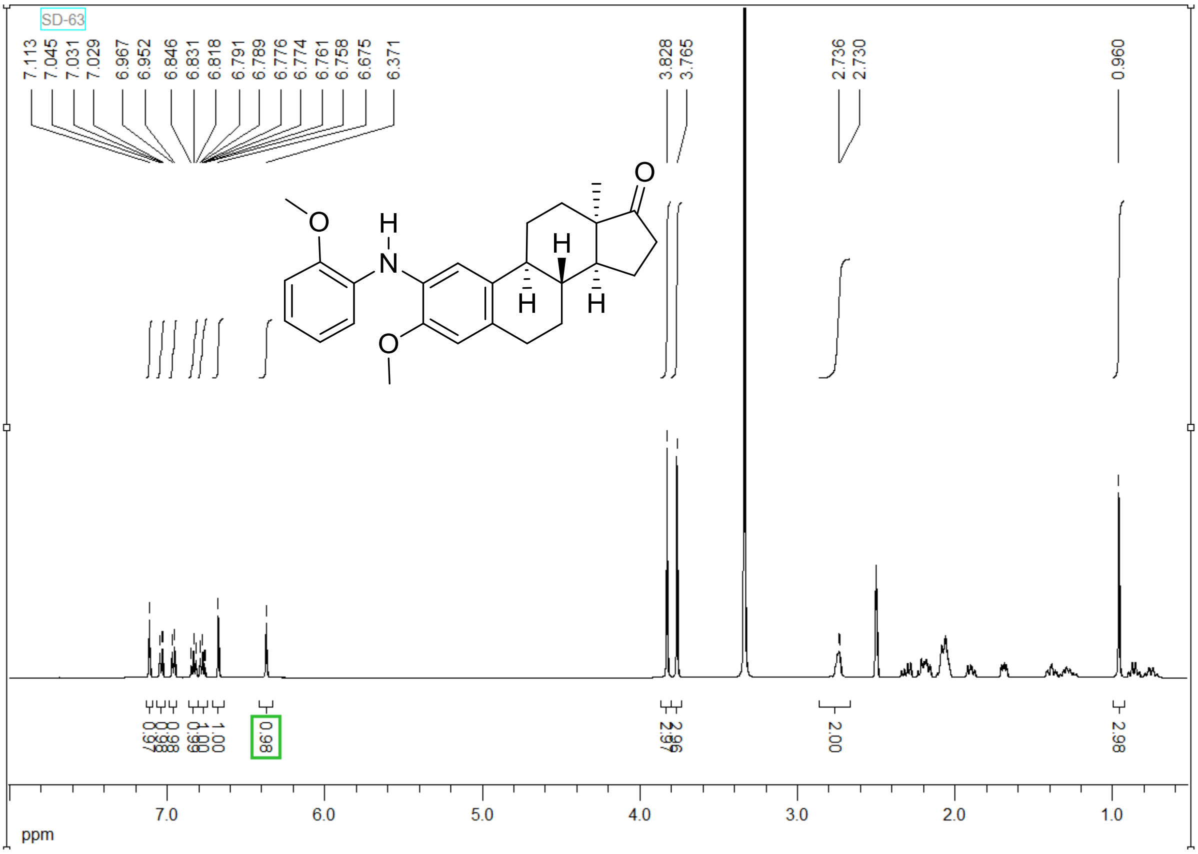
SD-63
7.113
7.045
7.031
7.029
6.967
6.952
6.846
6.831
6.818
6.791
6.789
6.776
6.774
6.761
6.758
6.675
6.371
3.828
3.765
2.736
2.730
0.960
0.98
2.00
2.98
7.0
6.0
5.0
4.0
3.0
2.0
1.0
ppm

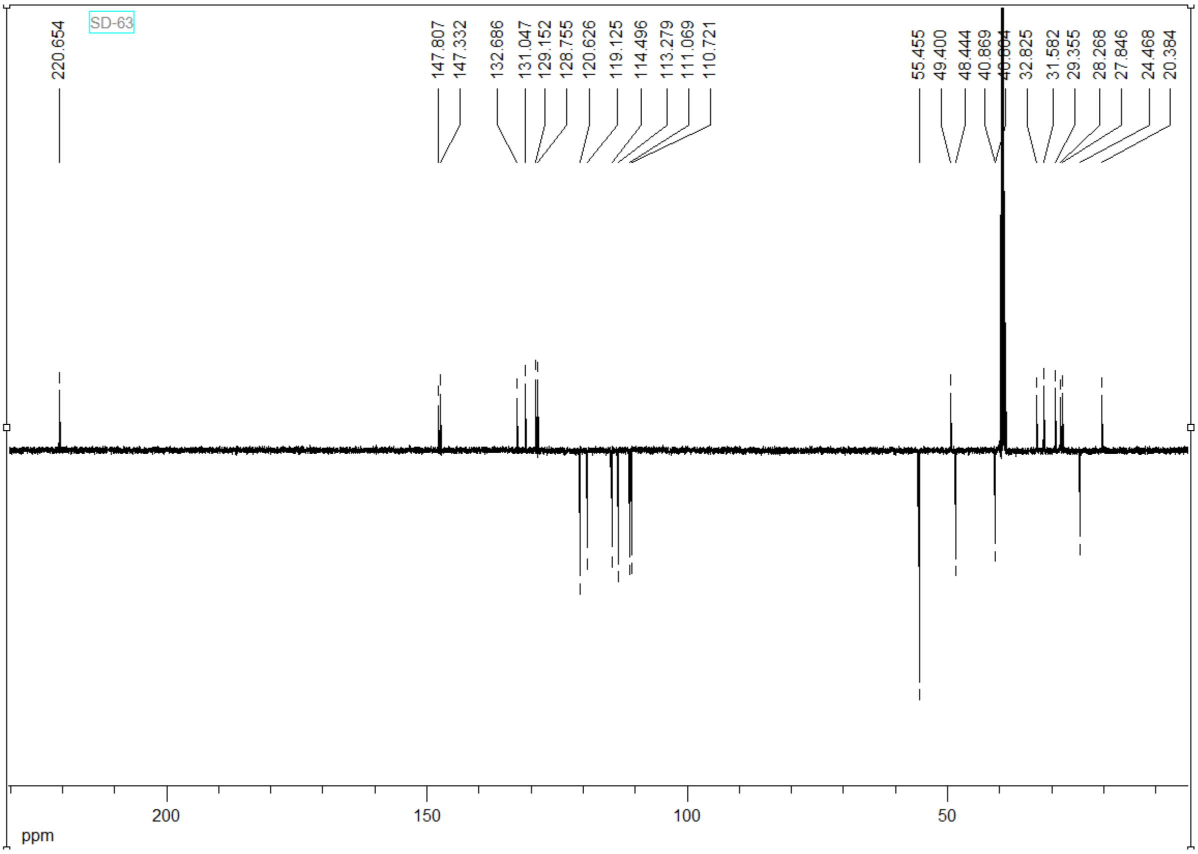
SD-63
220.654
147.807
147.332
132.686
131.047
129.152
128.755
120.626
119.125
114.496
113.279
111.069
110.721
55.455
49.400
48.444
40.869
32.825
31.582
29.355
28.268
27.846
24.468
20.384
200
150
100
50
ppm

7c

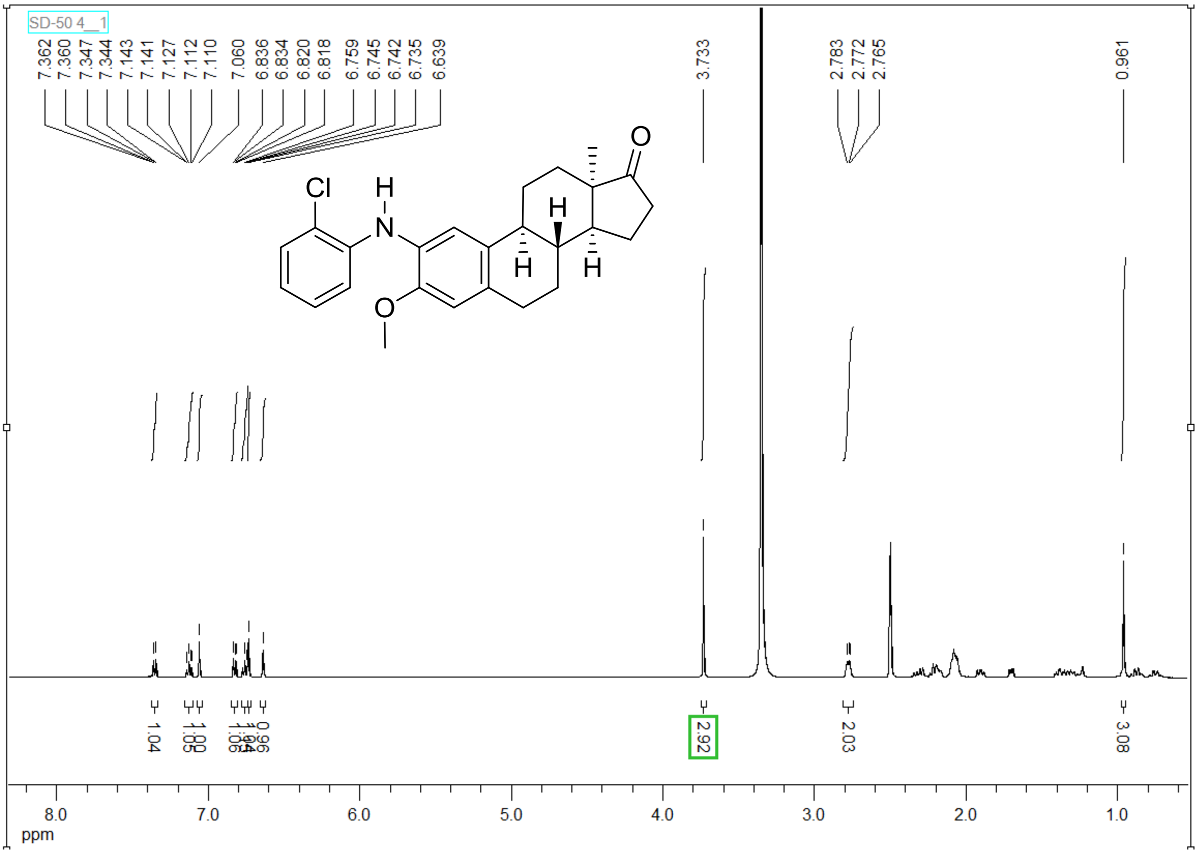

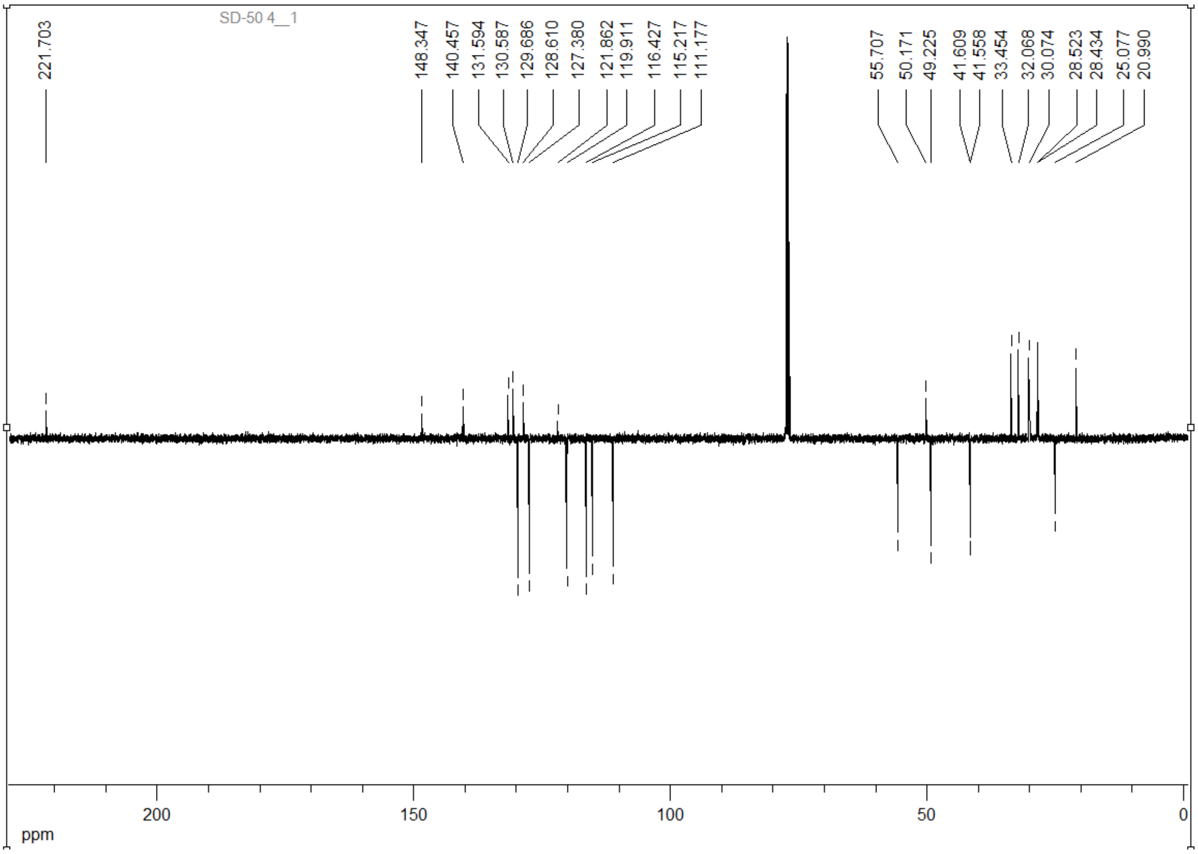

7d

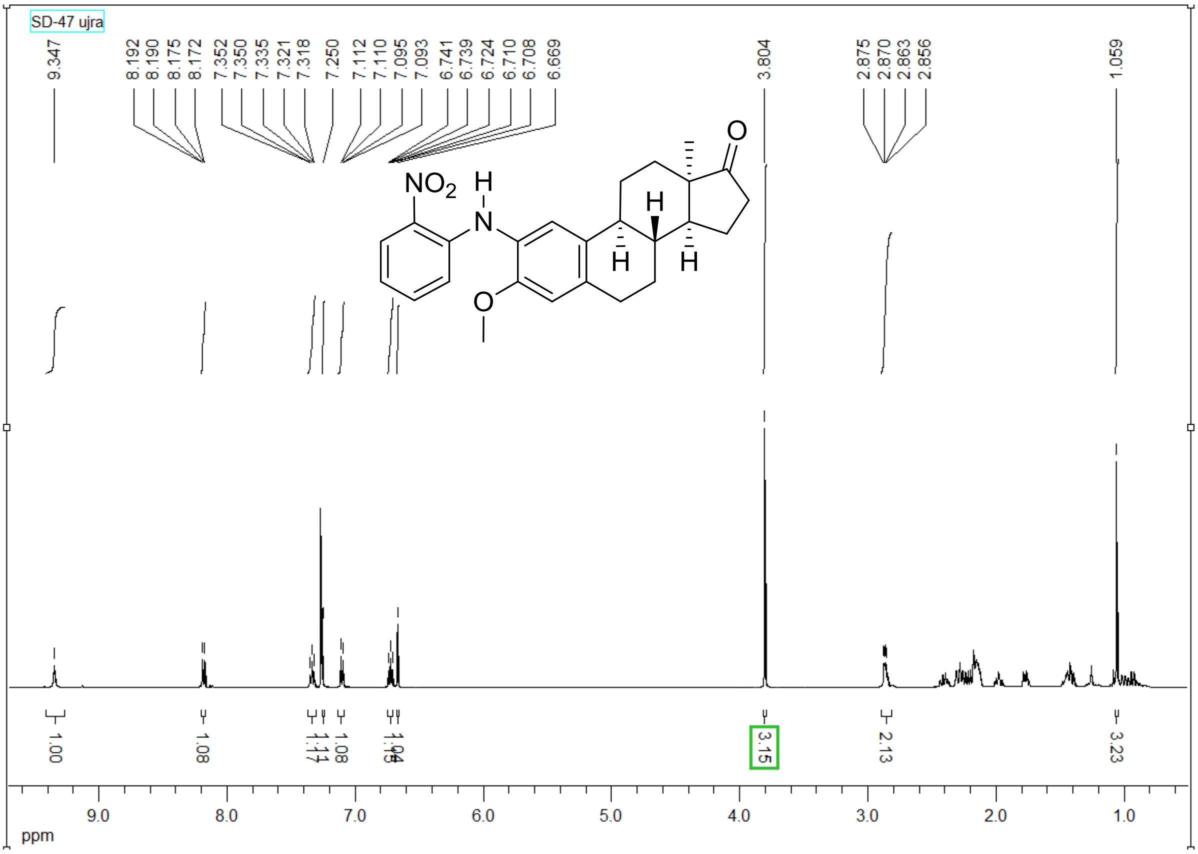
SD-47 ujra
9.347
8.192
8.190
8.175
8.172
7.352
7.350
7.335
7.321
7.318
7.250
7.112
7.110
7.095
7.093
6.741
6.739
6.724
6.710
6.708
6.669
3.804
2.875
2.870
2.863
2.856
1.059
NO2 H N O H H H O
1.00
1.08
1.17
1.11
1.08
1.04
3.15
2.13
3.23
9.0
8.0
7.0
6.0
5.0
4.0
3.0
2.0
1.0
ppm

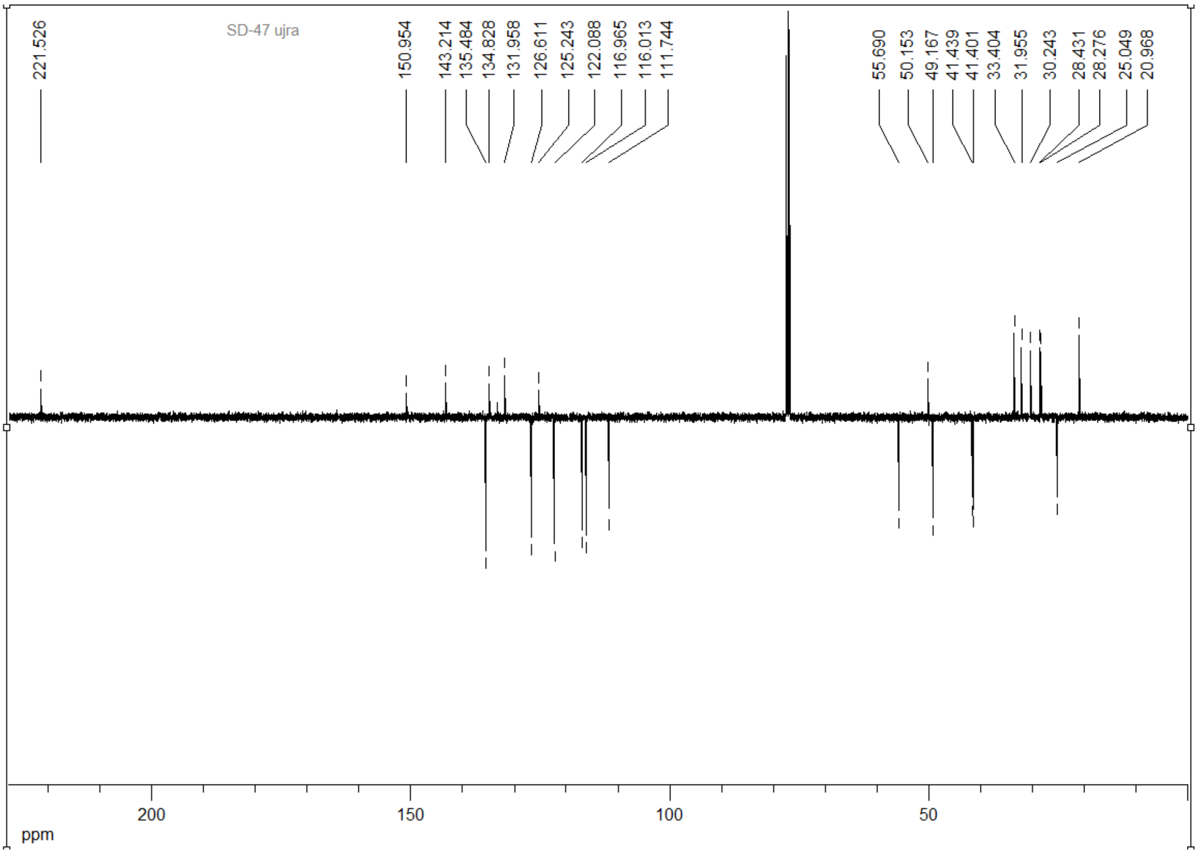
221.526
SD-47 ujra
150.954
143.214
135.484
134.828
131.958
126.611
125.243
122.088
116.965
116.013
111.744
55.690
50.153
49.167
41.439
41.401
33.404
31.955
30.243
28.431
28.276
25.049
20.968
200
150
100
50
ppm

8a

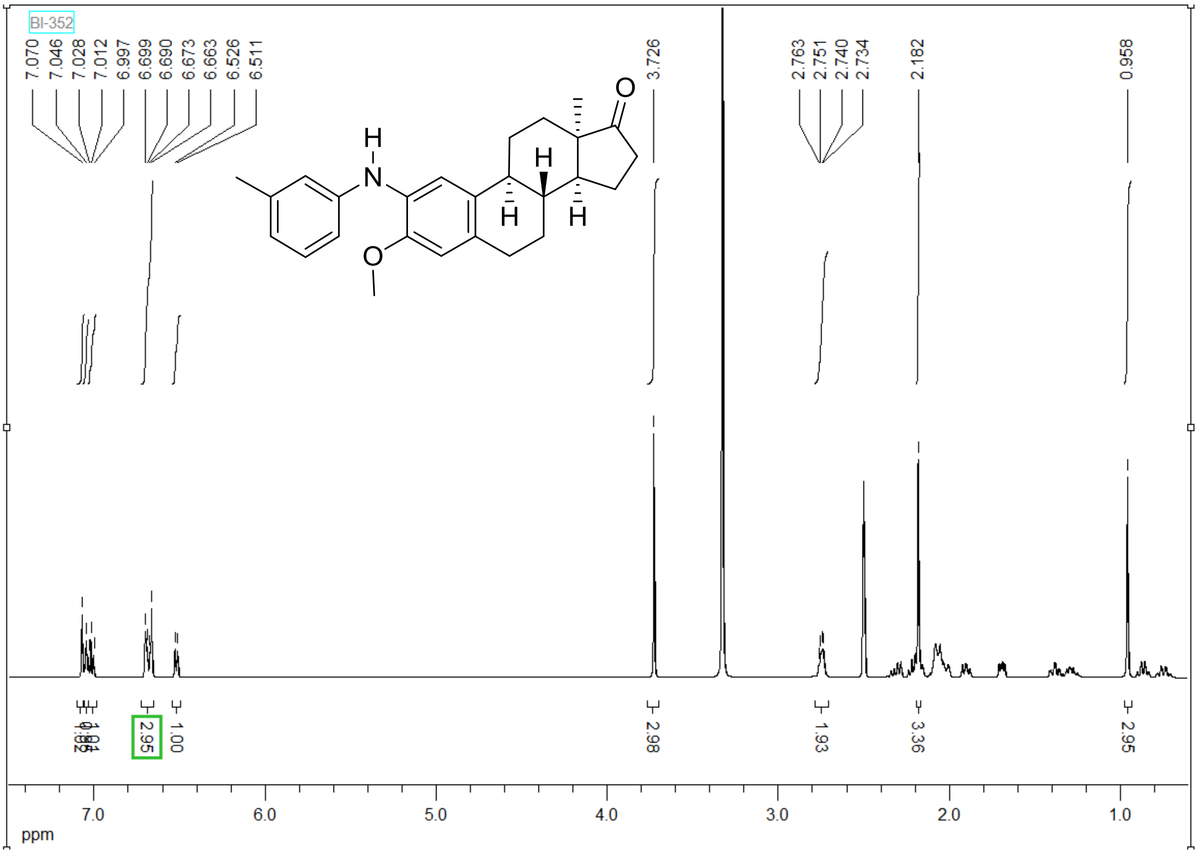
BI-352
7.070
7.046
7.028
7.012
6.997
6.699
6.690
6.673
6.663
6.526
6.511
3.726
2.763
2.751
2.740
2.734
2.182
0.958
2.95
1.00
2.98
1.93
3.36
2.95
7.0
6.0
5.0
4.0
3.0
2.0
1.0
ppm

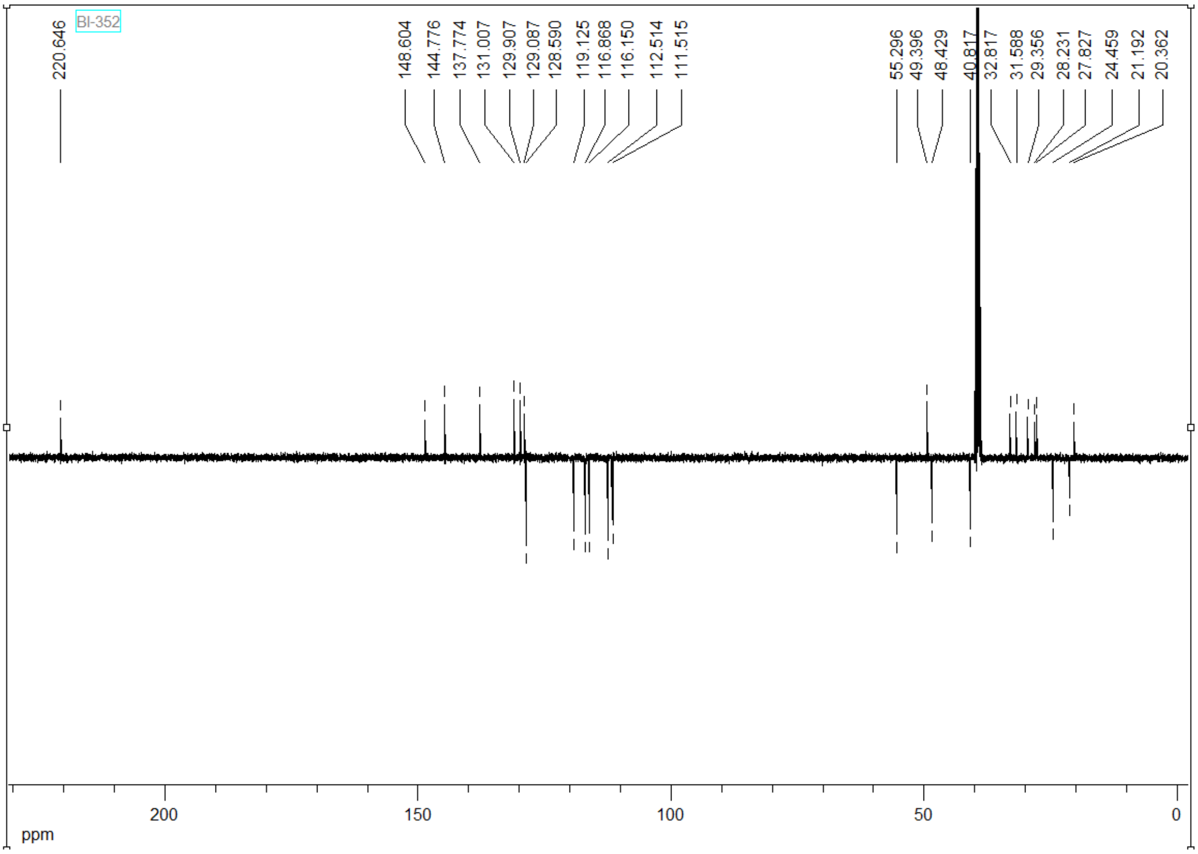
BI-352
220.646
148.604
144.776
137.774
131.007
129.907
129.087
128.590
119.125
116.868
116.150
112.514
111.515
55.296
49.396
48.429
40.817
32.817
31.588
29.356
28.231
27.827
24.459
21.192
20.362
200
150
100
50
0
ppm

8b

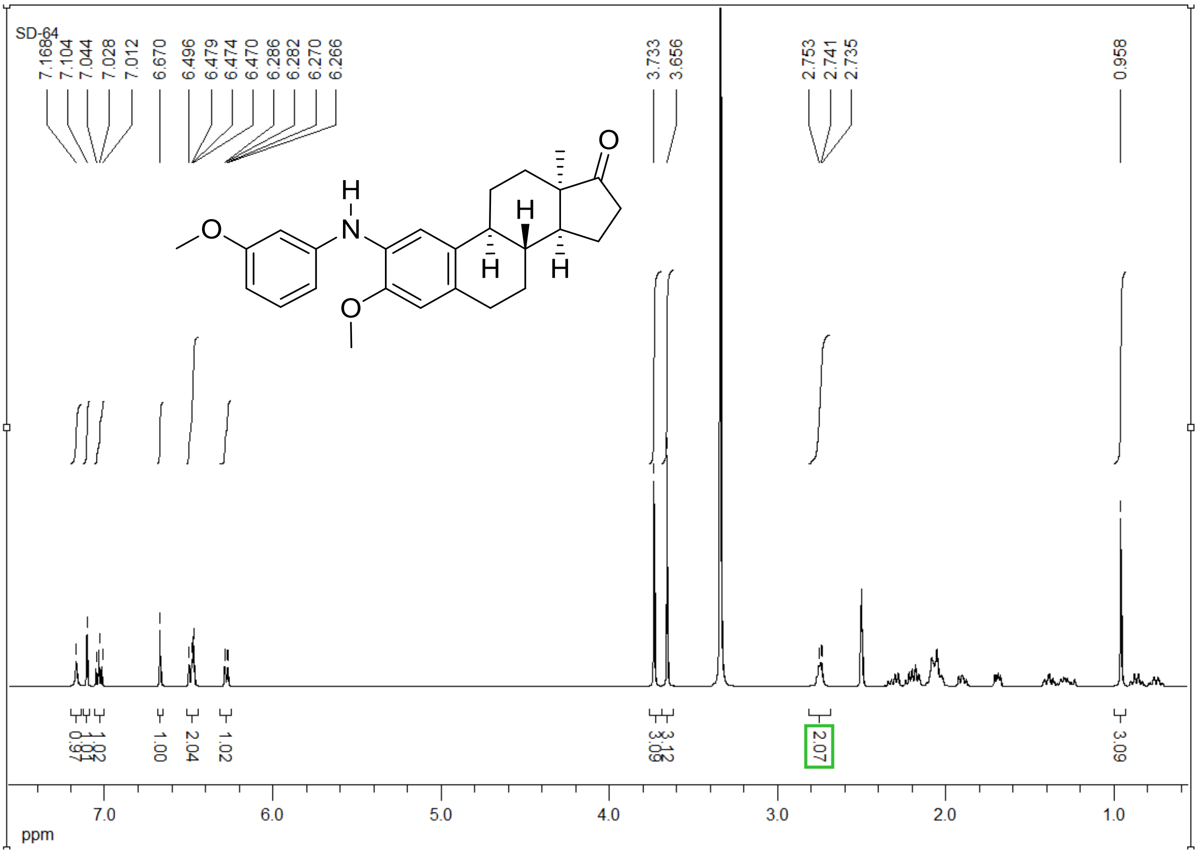

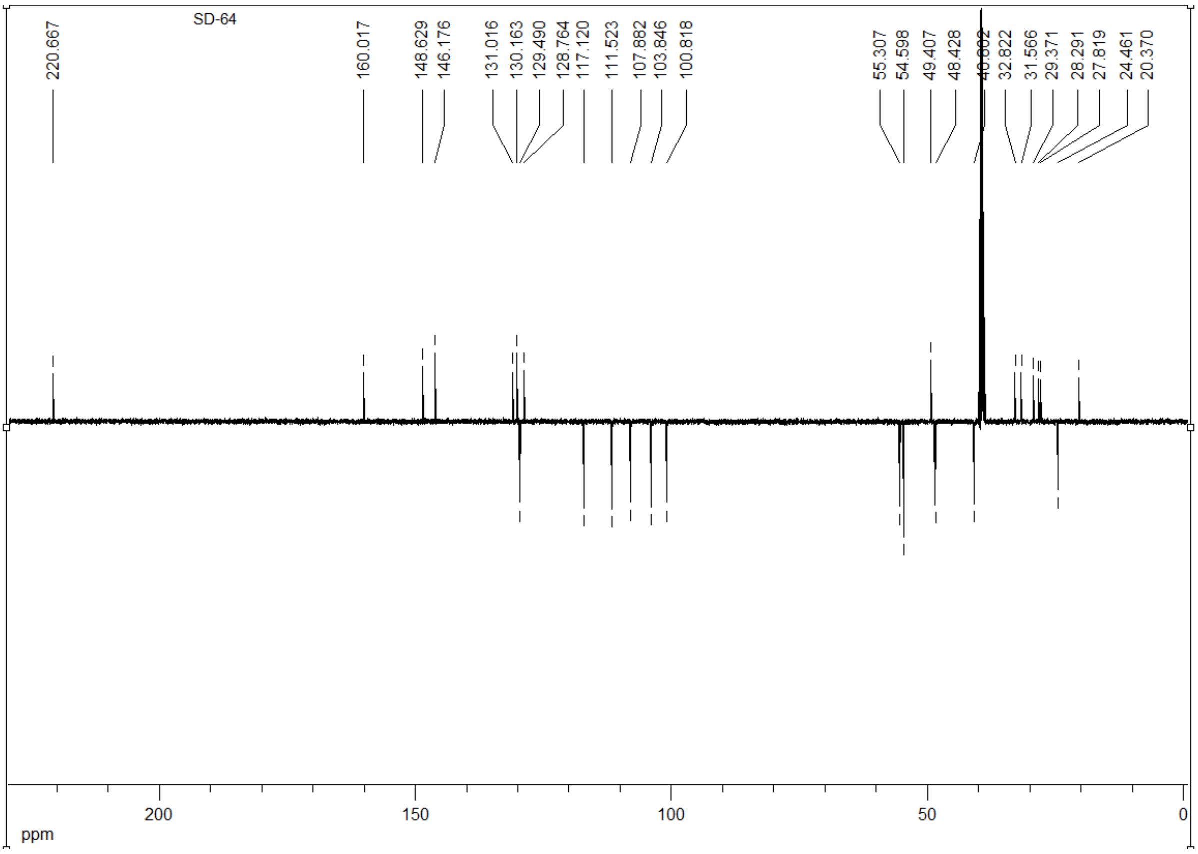

8c

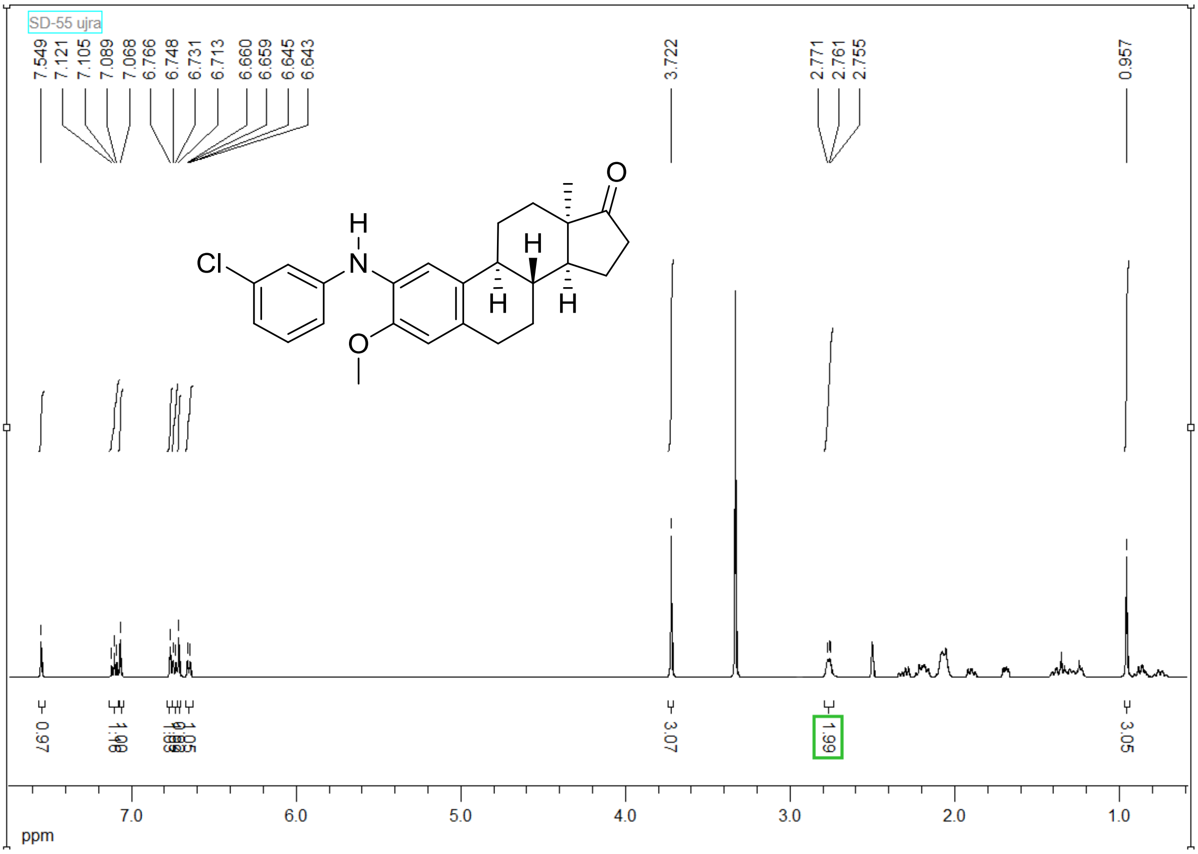
SD-55 ujra
7.549
7.121
7.105
7.089
7.068
6.766
6.748
6.731
6.713
6.660
6.659
6.645
6.643
3.722
2.771
2.761
2.755
0.957
Cl
H
N
O
H
H
H
O
0.97
3.07
1.99
3.05
7.0
6.0
5.0
4.0
3.0
2.0
1.0
ppm

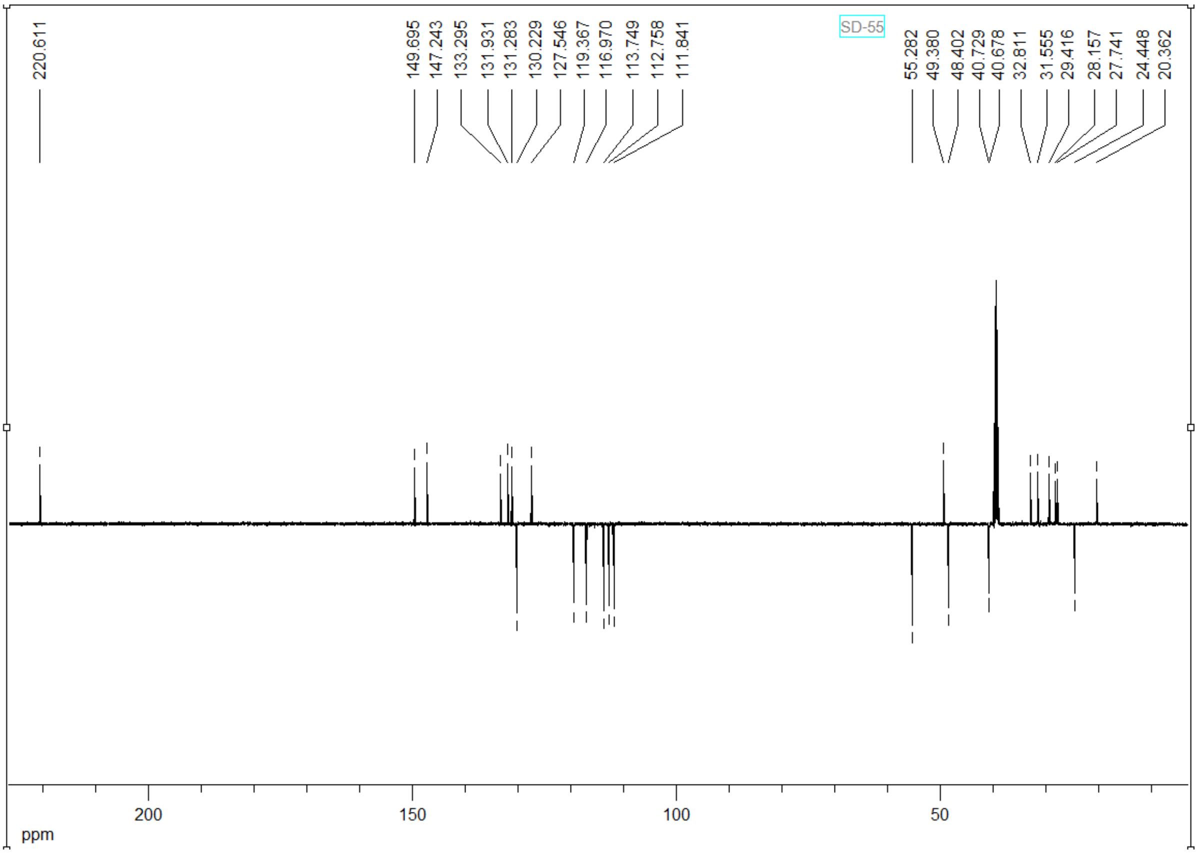
220.611
149.695
147.243
133.295
131.931
131.283
130.229
127.546
119.367
116.970
113.749
112.758
111.841
SD-55
55.282
49.380
48.402
40.729
40.678
32.811
31.555
29.416
28.157
27.741
24.448
20.362
200
150
100
50
ppm

8d

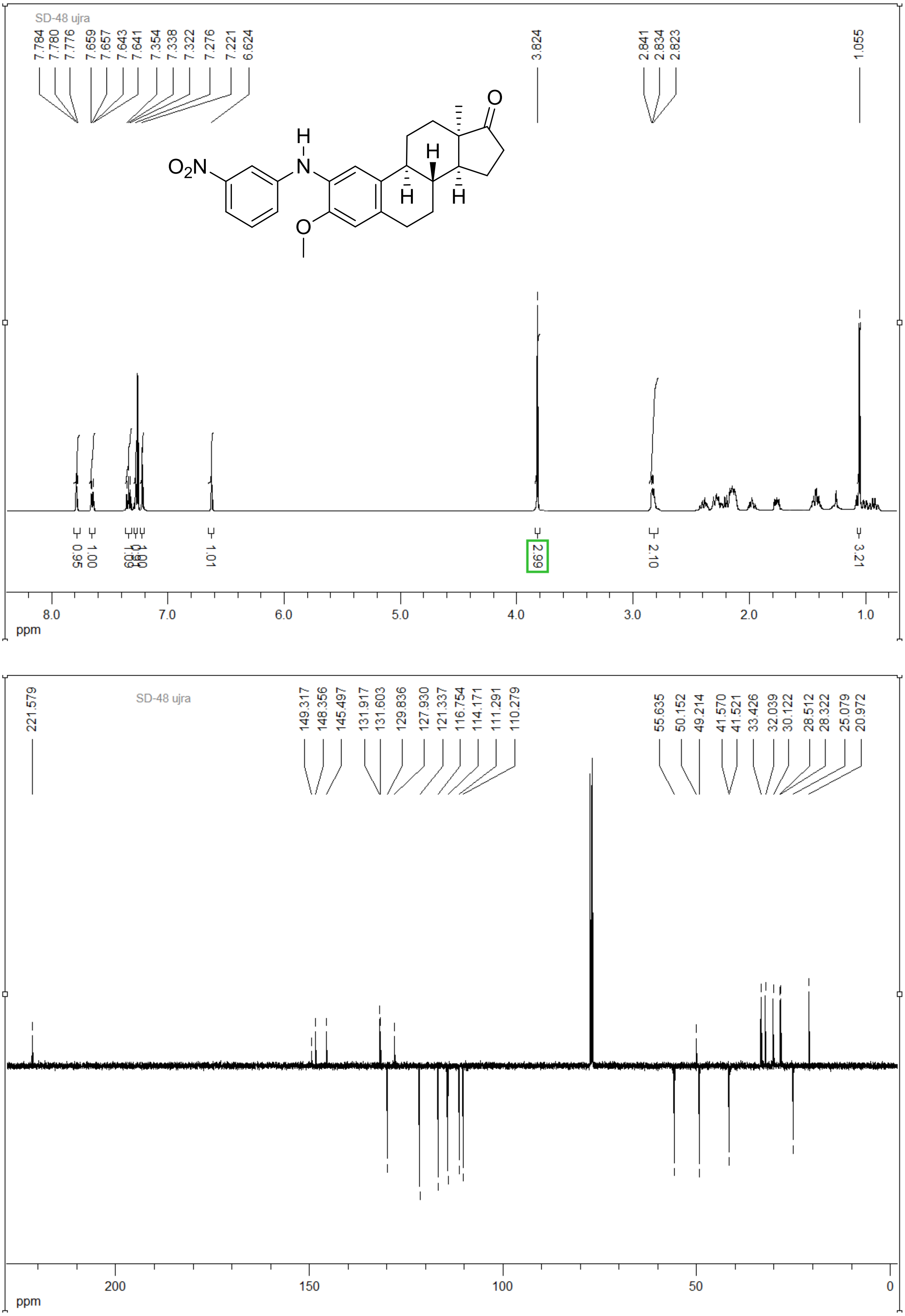
SD-48 ujra
7.784
7.780
7.776
7.659
7.657
7.643
7.641
7.354
7.338
7.322
7.276
7.221
6.624
3.824
2.841
2.834
2.823
1.055
0.95
1.00
1.01
2.99
2.10
3.21
8.0
7.0
6.0
5.0
4.0
3.0
2.0
1.0
ppm
221.579
SD-48 ujra
149.317
148.356
145.497
131.917
131.603
129.836
127.930
121.337
116.754
114.171
111.291
110.279
55.635
50.152
49.214
41.570
41.521
33.426
32.039
30.122
28.512
28.322
25.079
20.972
200
150
100
50
0
ppm

9a

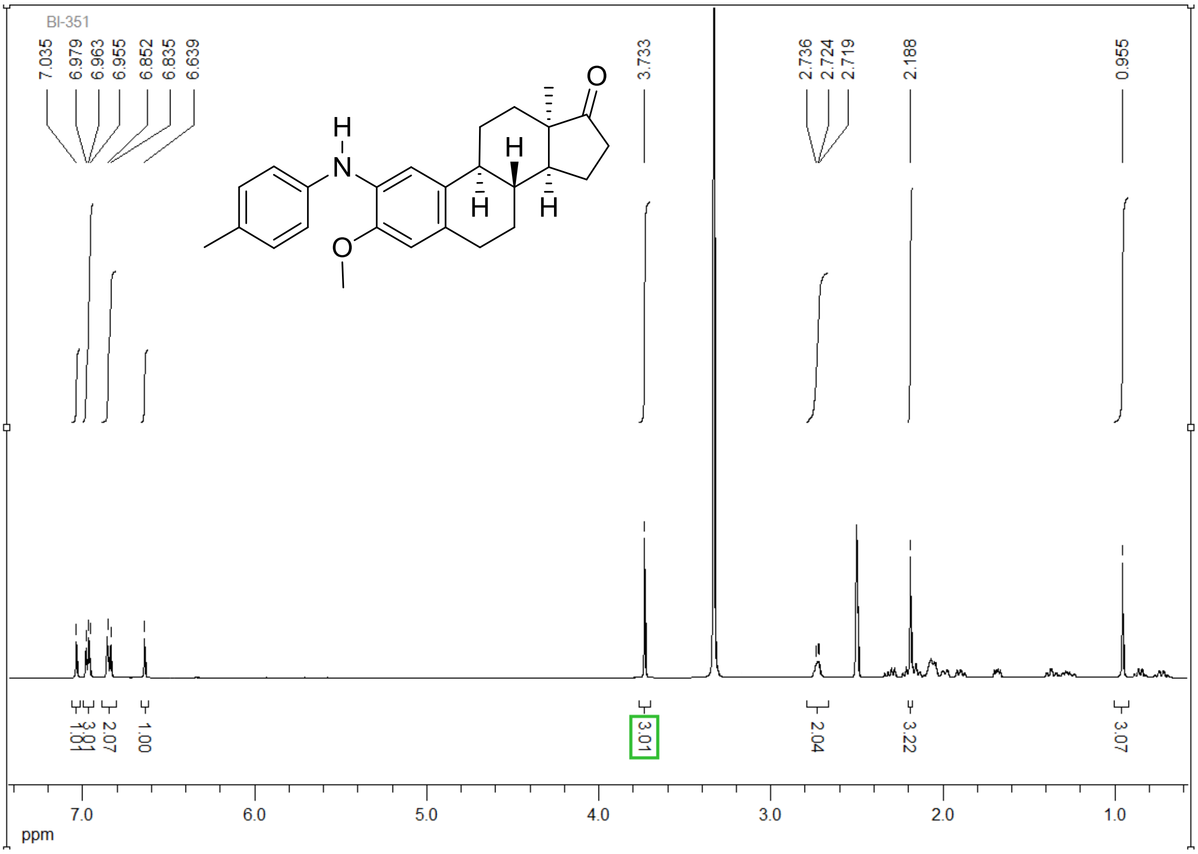
BI-351
7.035
6.979
6.963
6.955
6.852
6.835
6.639
3.733
2.736
2.724
2.719
2.188
0.955
1.81
3.01
2.07
1.00
3.01
2.04
3.22
3.07
7.0
6.0
5.0
4.0
3.0
2.0
1.0
ppm

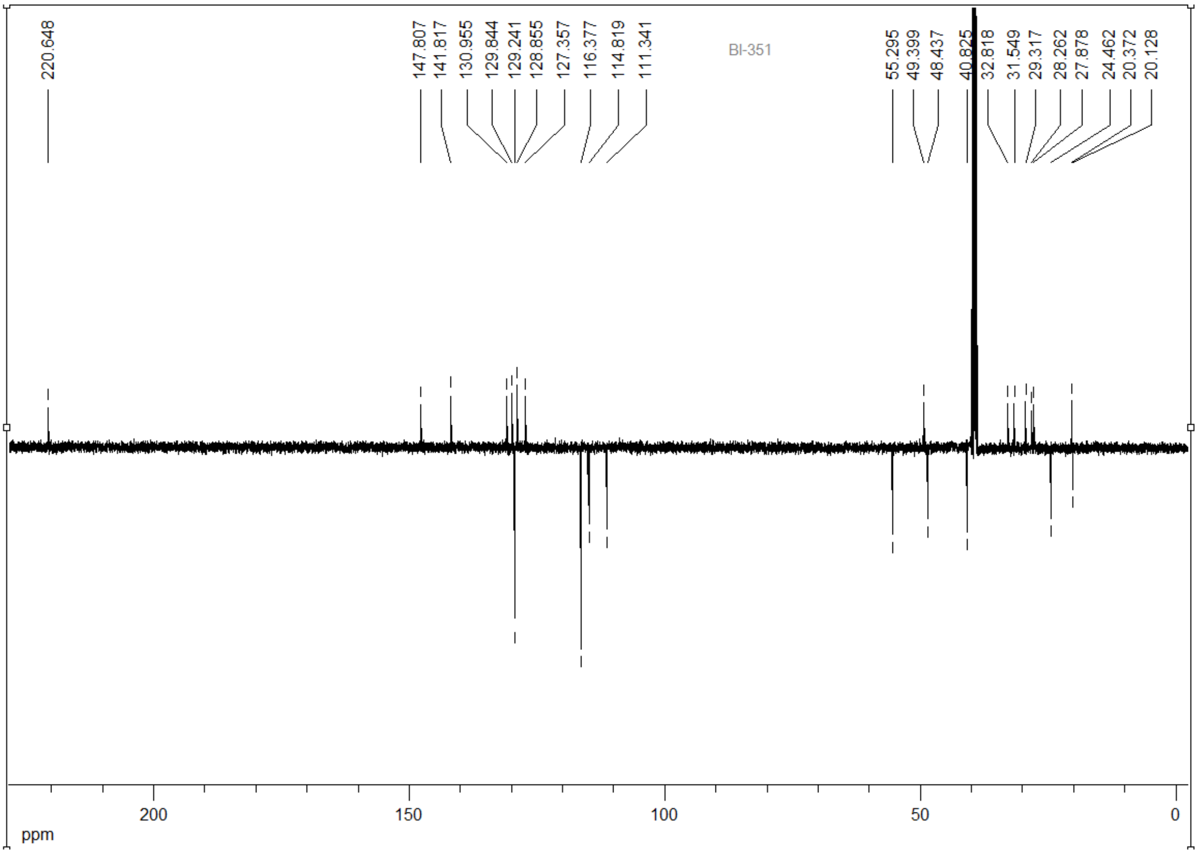
220.648
147.807
141.817
130.955
129.844
129.241
128.855
127.357
116.377
114.819
111.341
BI-351
55.295
49.399
48.437
40.825
32.818
31.549
29.317
28.262
27.878
24.462
20.372
20.128
200
150
100
50
0
ppm

9b

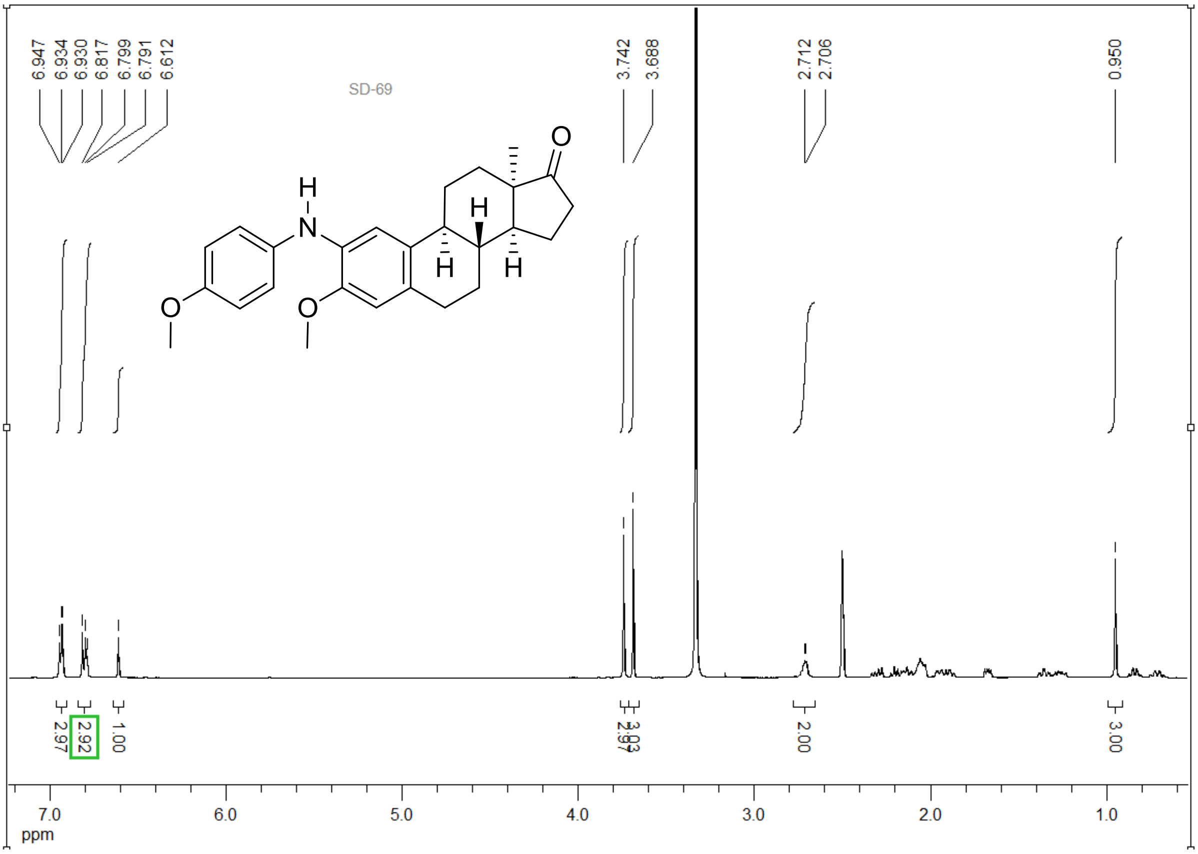
SD-69
6.947
6.934
6.930
6.817
6.799
6.791
6.612
3.742
3.688
2.712
2.706
0.950
2.97
2.92
1.00
2.97
3.03
2.00
3.00
7.0
6.0
5.0
4.0
3.0
2.0
1.0
ppm

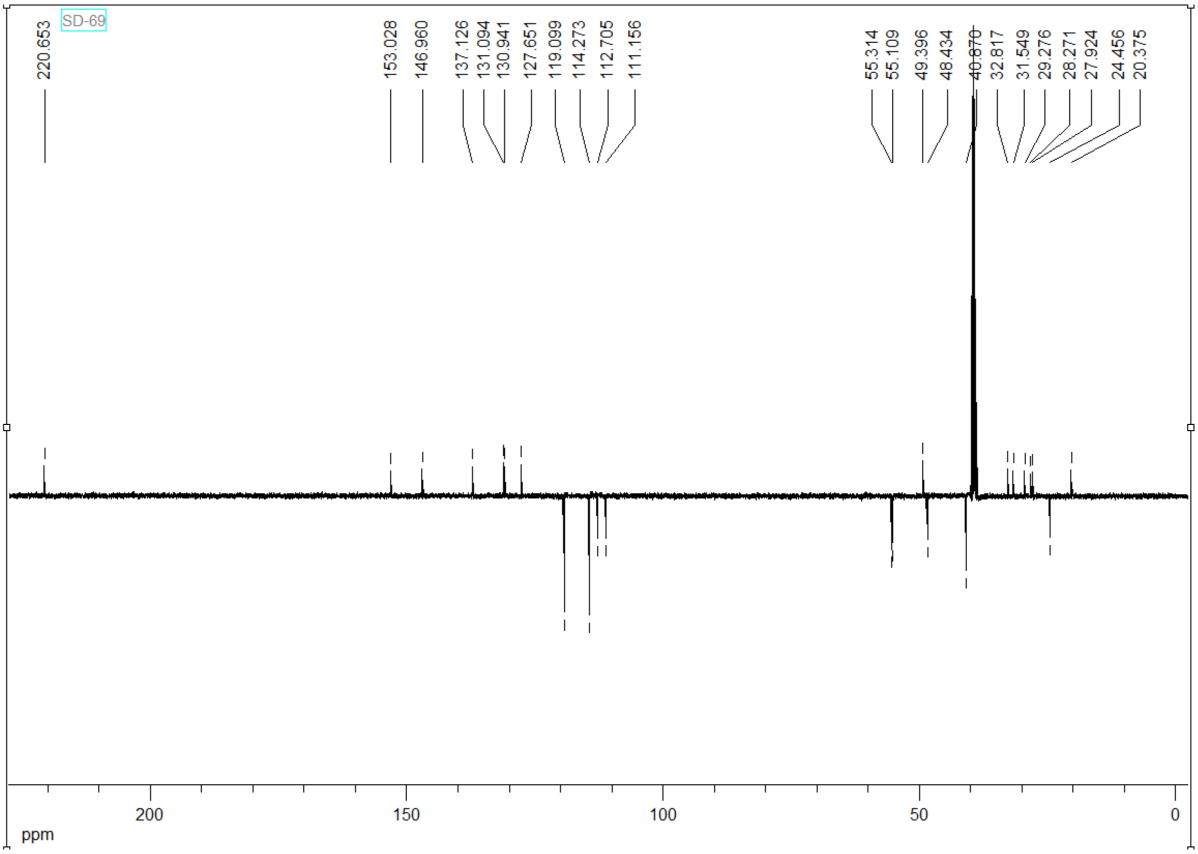
SD-69
220.653
153.028
146.960
137.126
131.094
130.941
127.651
119.099
114.273
112.705
111.156
55.314
55.109
49.396
48.434
40.870
32.817
31.549
29.276
28.271
27.924
24.456
20.375
200
150
100
50
0
ppm

9c

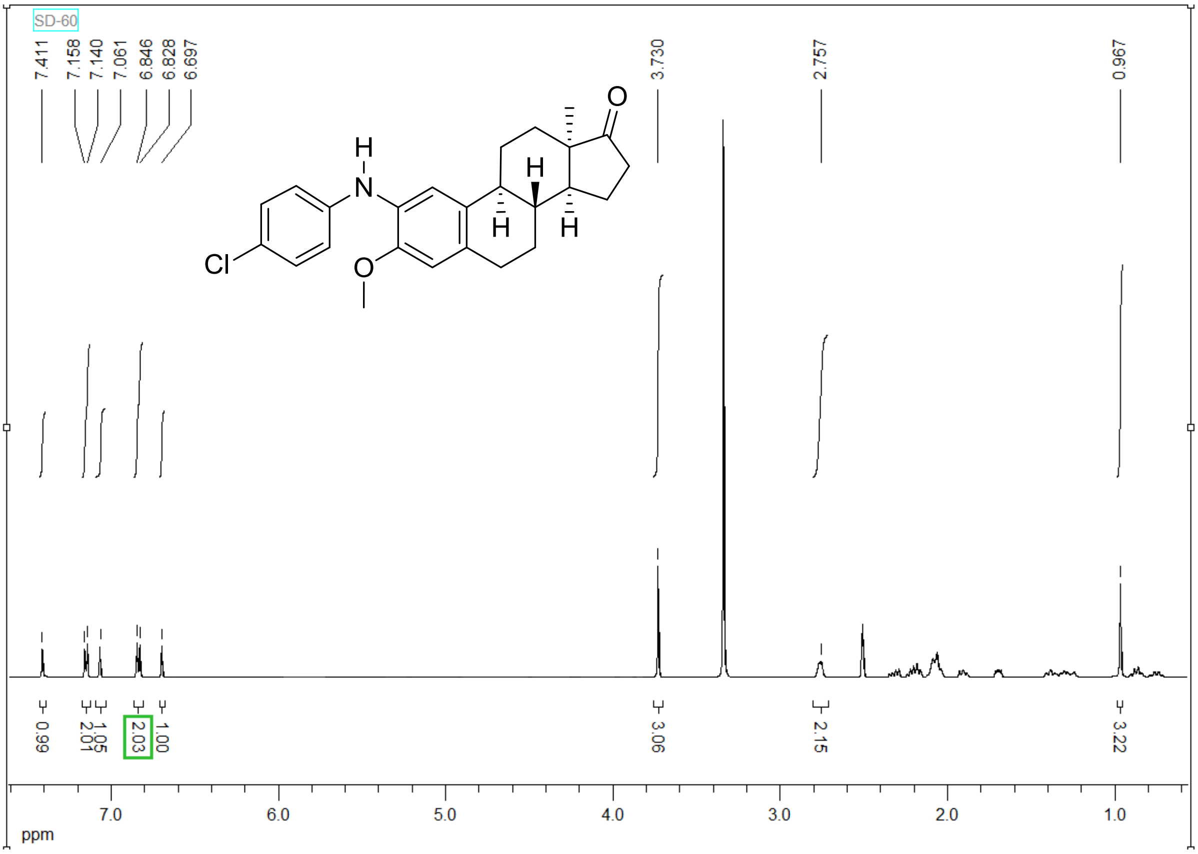
SD-60
7.411
7.158
7.140
7.061
6.846
6.828
6.697
3.730
2.757
0.967
Cl
H
N
O
H
H
H
O
0.99
2.01
1.05
2.03
1.00
3.06
2.15
3.22
7.0
6.0
5.0
4.0
3.0
2.0
1.0
ppm

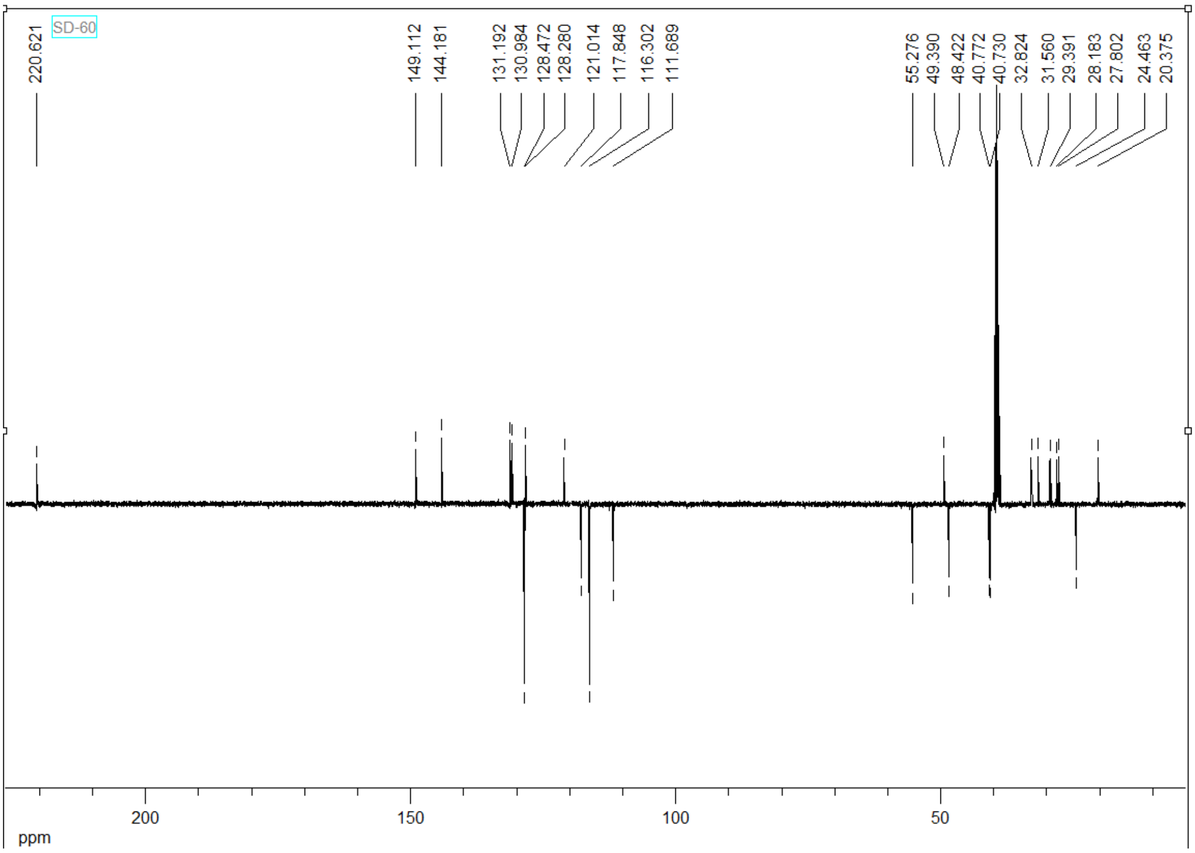
SD-60
220.621
149.112
144.181
131.192
130.984
128.472
128.280
121.014
117.848
116.302
111.689
55.276
49.390
48.422
40.772
40.730
32.824
31.560
29.391
28.183
27.802
24.463
20.375
200
150
100
50
ppm

9d

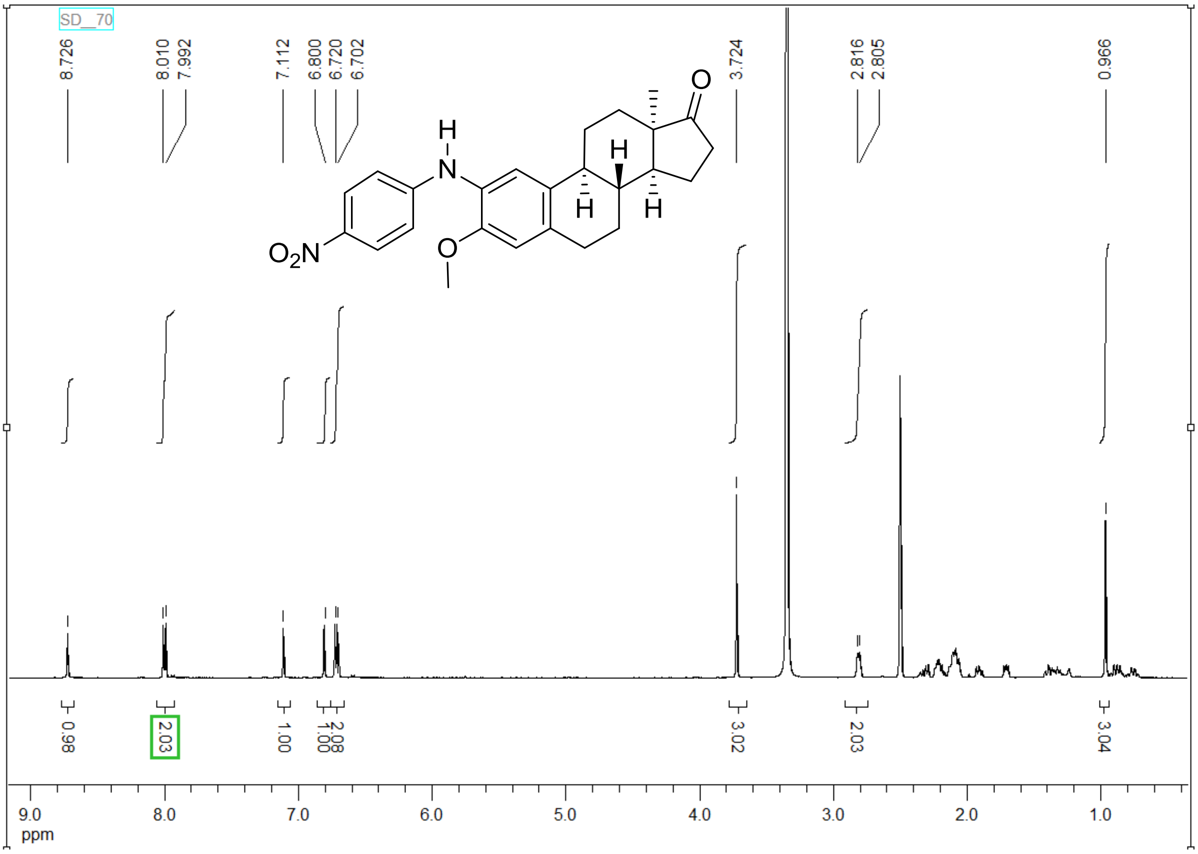

SD_70
8.726
8.010
7.992
7.112
6.800
6.720
6.702
3.724
2.816
2.805
0.966
O
H
N
H
H
H
O$_2$N
O
0.98
2.03
1.00
1.00
2.08
3.02
2.03
3.04
9.0
8.0
7.0
6.0
5.0
4.0
3.0
2.0
1.0
ppm

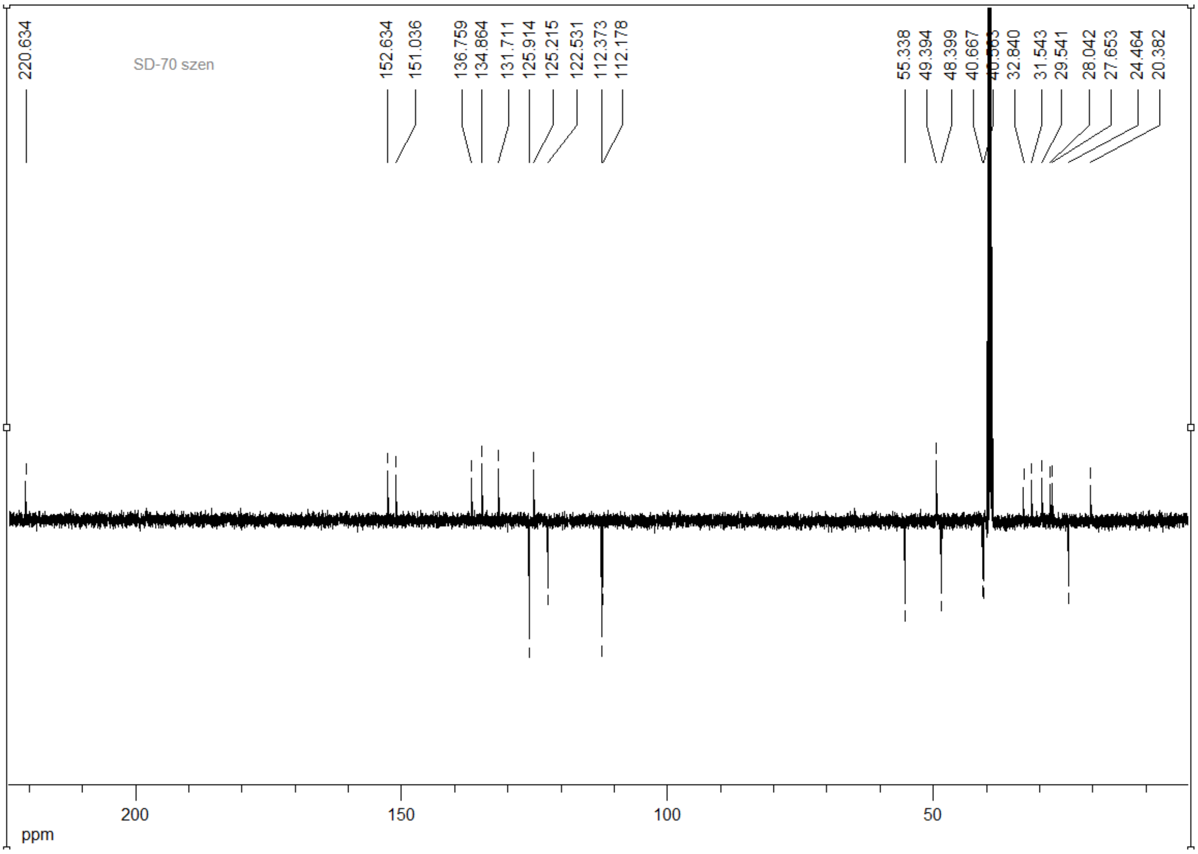

220.634
SD-70 szen
152.634
151.036
136.759
134.864
131.711
125.914
125.215
122.531
112.373
112.178
55.338
49.394
48.399
40.667
32.840
31.543
29.541
28.042
27.653
24.464
20.382
200
150
100
50
ppm

10

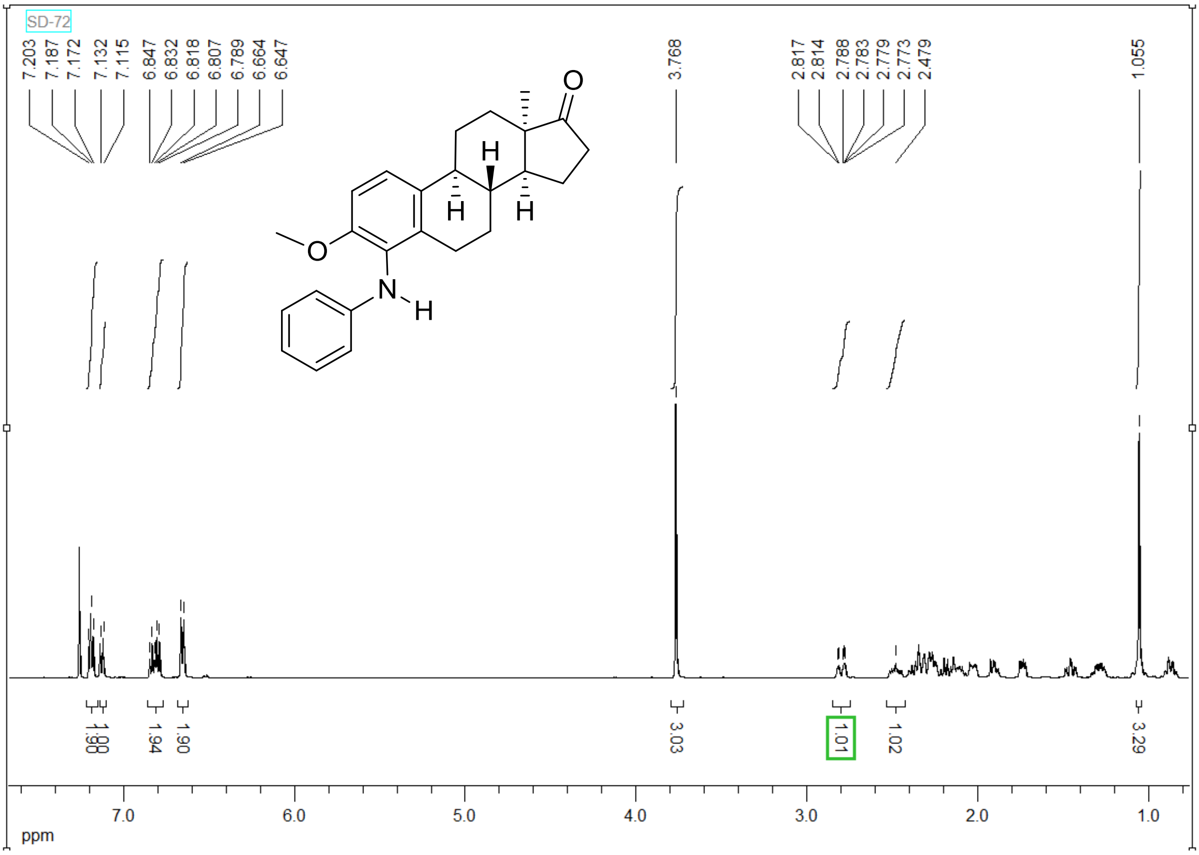
SD-72
7.203
7.187
7.172
7.132
7.115
6.847
6.832
6.818
6.807
6.789
6.664
6.647
3.768
2.817
2.814
2.788
2.783
2.779
2.773
2.479
1.055
O
H
H
H
O
N
H
1.98
1.94
1.90
3.03
1.01
1.02
3.29
7.0
6.0
5.0
4.0
3.0
2.0
1.0
ppm

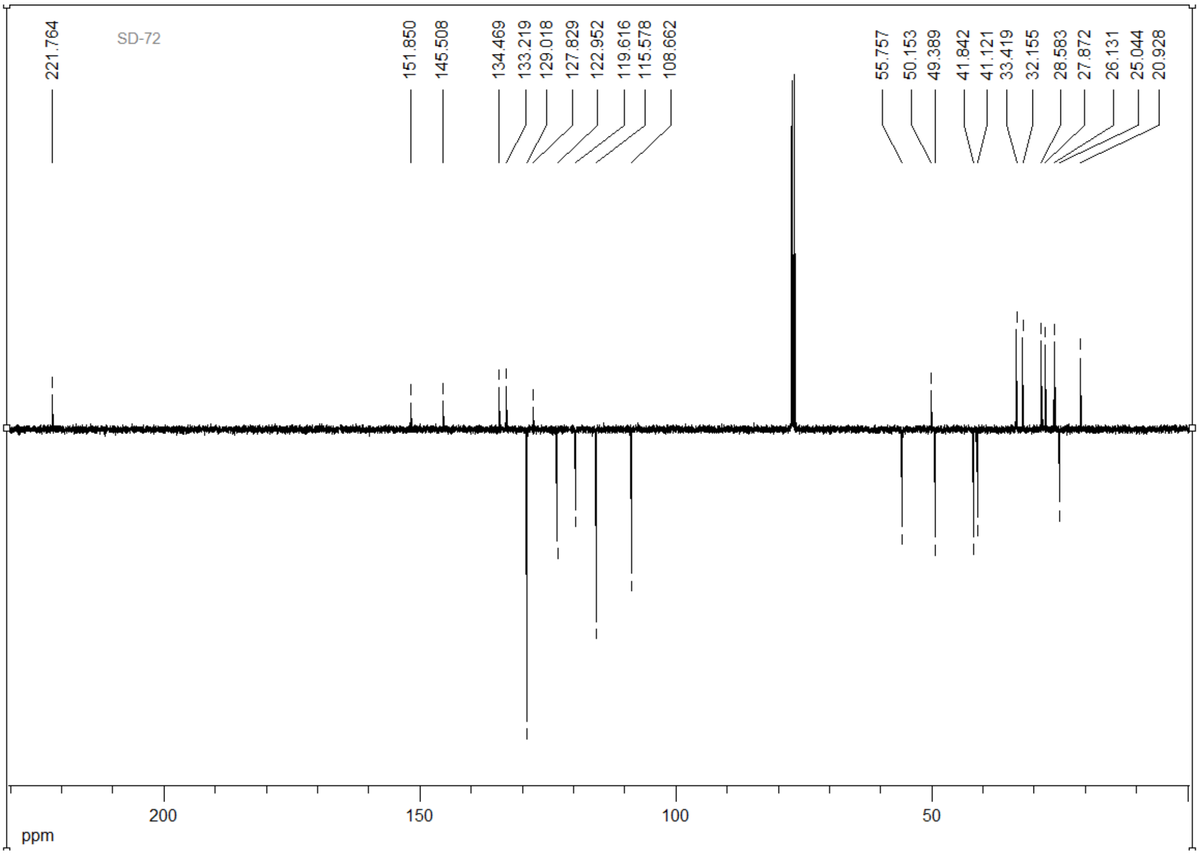
221.764
SD-72
151.850
145.508
134.469
133.219
129.018
127.829
122.952
119.616
115.578
108.662
55.757
50.153
49.389
41.842
41.121
33.419
32.155
28.583
27.872
26.131
25.044
20.928
200
150
100
50
ppm

11

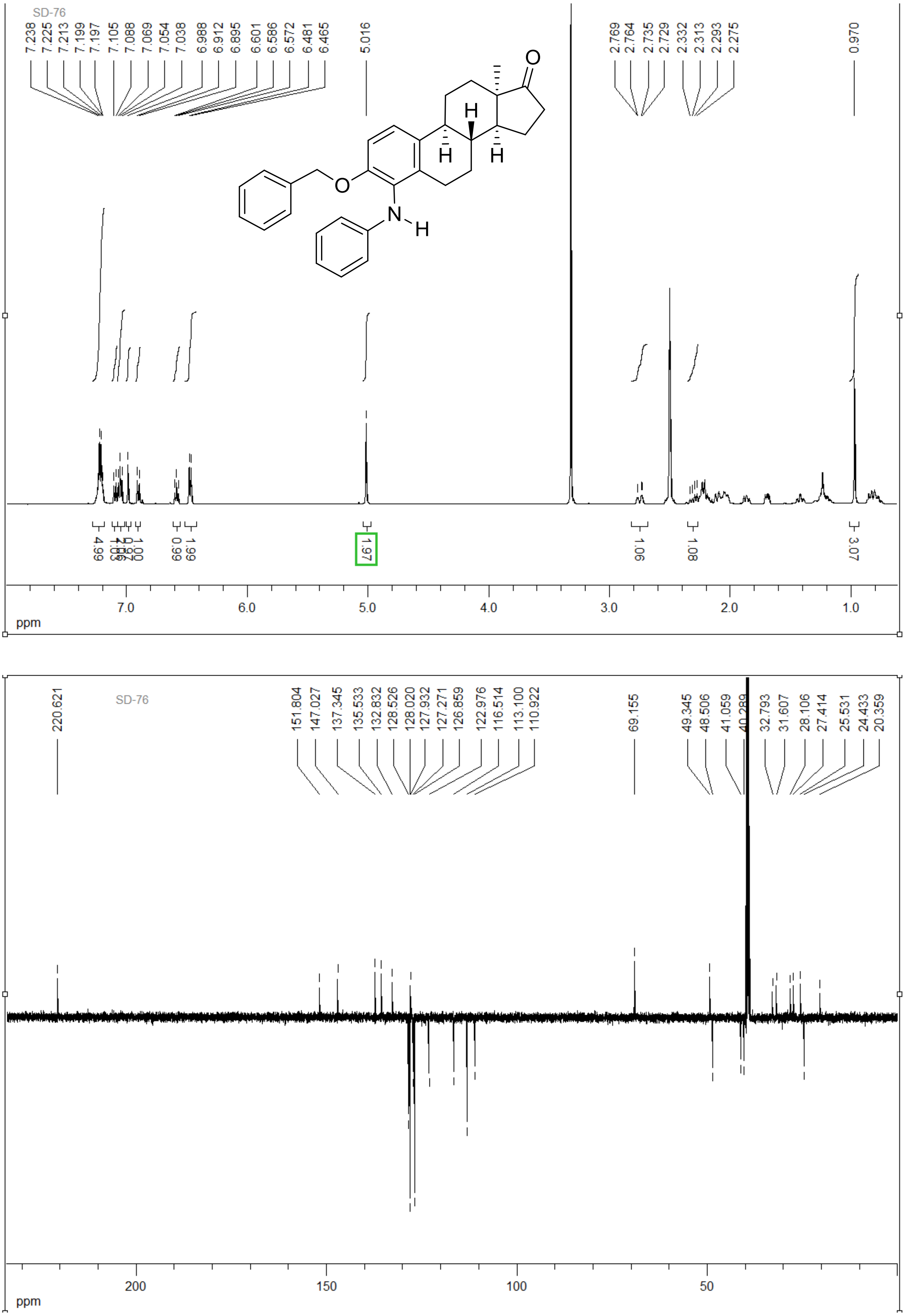

12

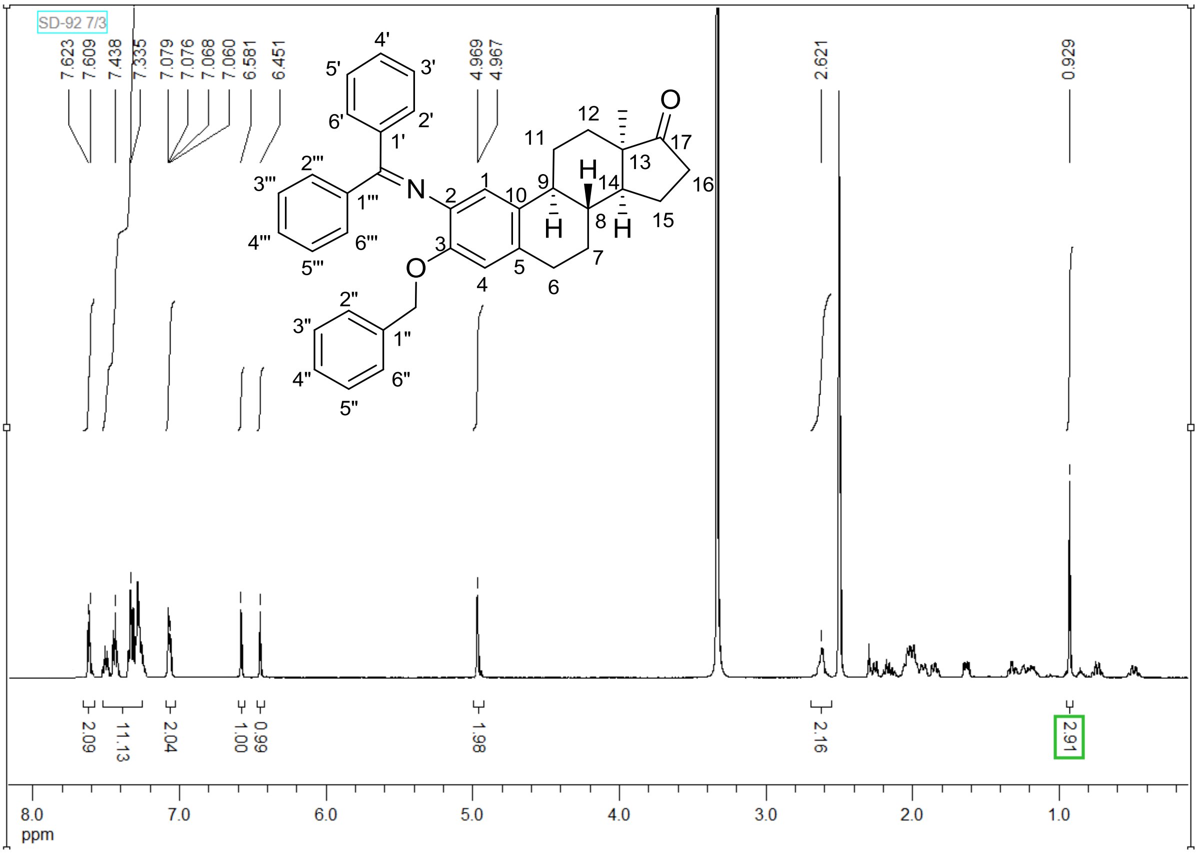

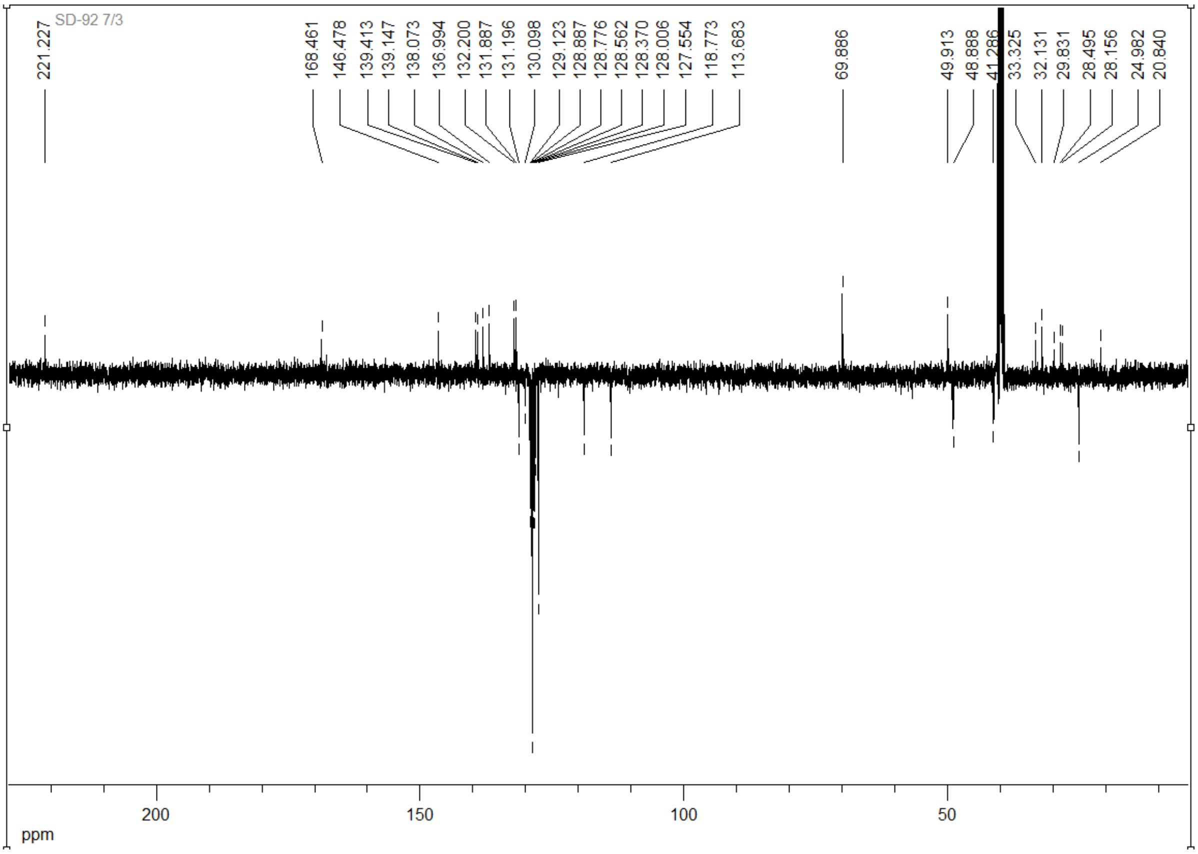

13

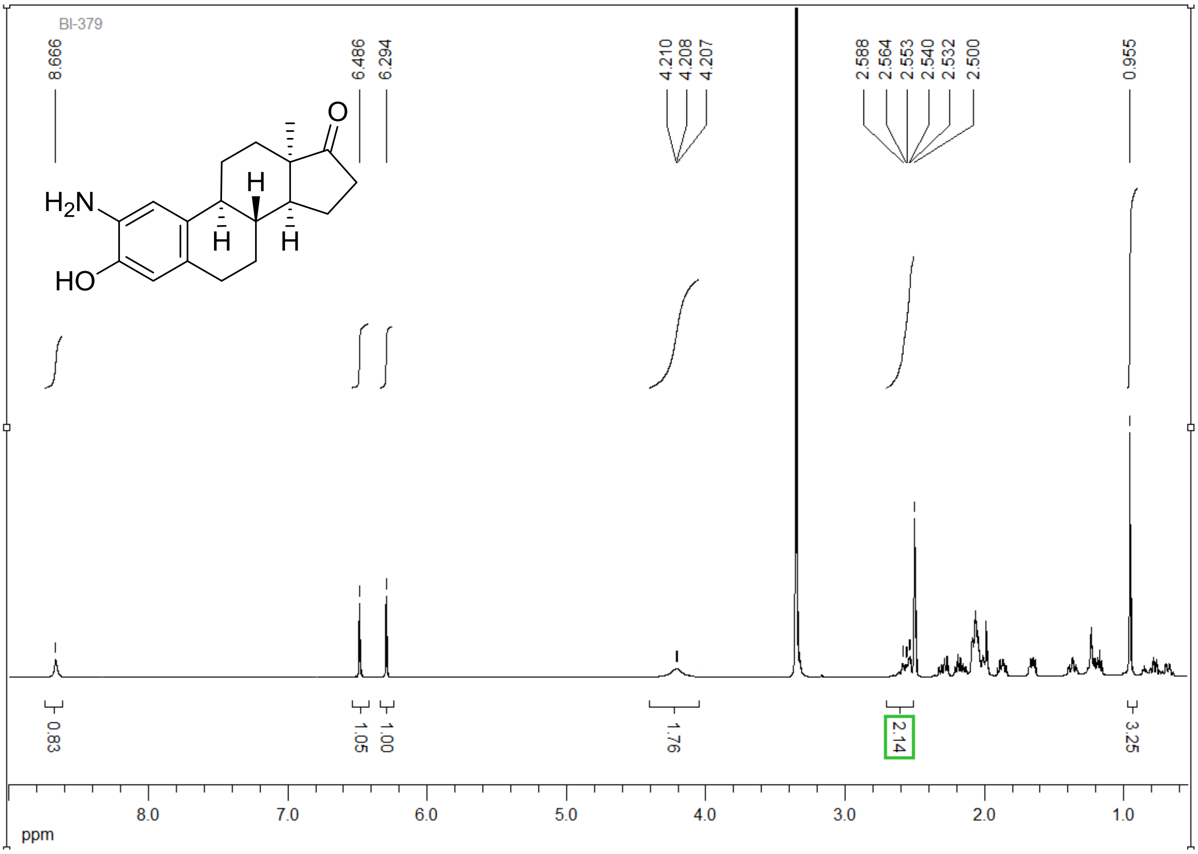
BI-379
8.666
6.486
6.294
4.210
4.208
4.207
2.588
2.564
2.553
2.540
2.532
2.500
0.955
H2N
HO
O
H
H
H
0.83
1.05
1.00
1.76
2.14
3.25
8.0
7.0
6.0
5.0
4.0
3.0
2.0
1.0
ppm

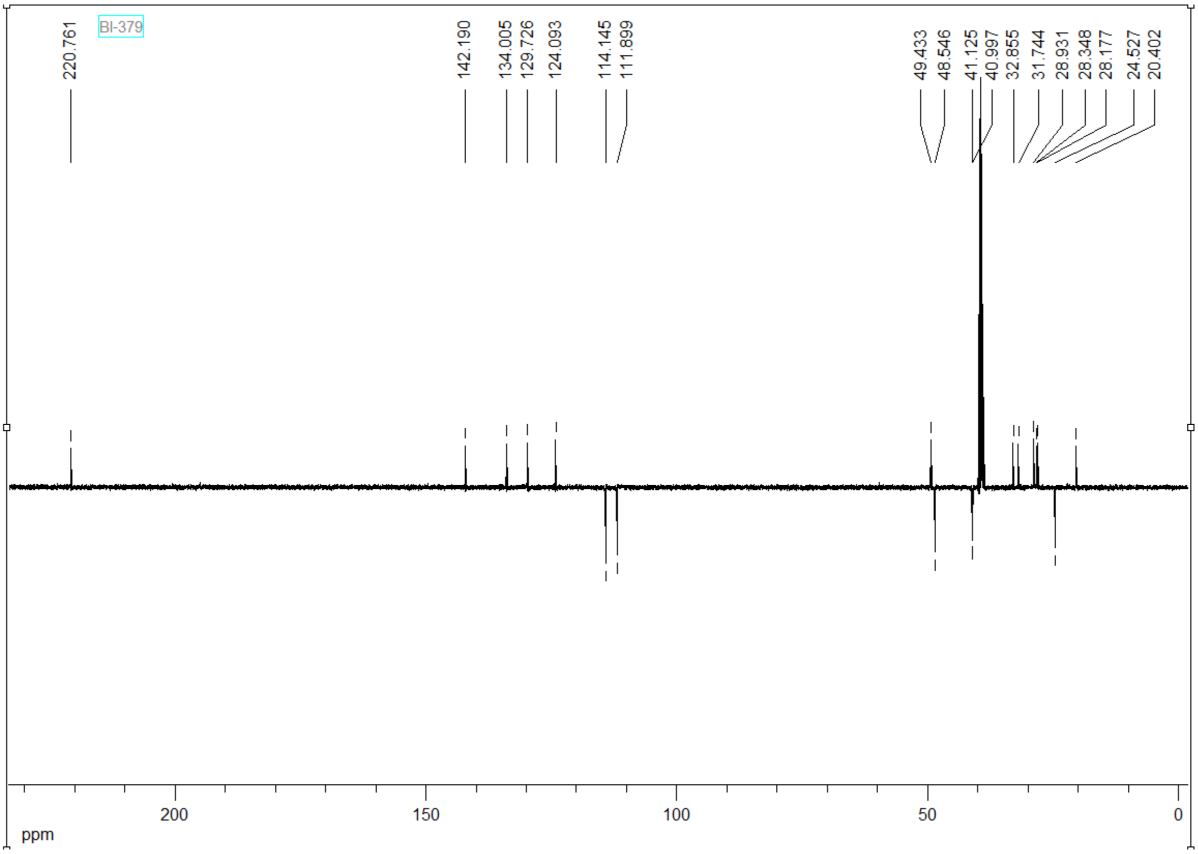
220.761
BI-379
142.190
134.005
129.726
124.093
114.145
111.899
49.433
48.546
41.125
40.997
32.855
31.744
28.931
28.348
28.177
24.527
20.402
200
150
100
50
0
ppm
